# Zerumbone Modulates α_2A_-Adrenergic, TRPV1, and NMDA NR2B Receptors Plasticity in CCI-Induced Neuropathic Pain *In Vivo* and LPS-Induced SH-SY5Y Neuroblastoma *In Vitro* Models

**DOI:** 10.3389/fphar.2020.00092

**Published:** 2020-03-04

**Authors:** Jasmine Siew Min Chia, Noor Aishah Mohammed Izham, Ahmad Akira Omar Farouk, Mohd Roslan Sulaiman, Sanam Mustafa, Mark R. Hutchinson, Enoch Kumar Perimal

**Affiliations:** ^1^Department of Biomedical Science, Faculty of Medicine and Health Sciences, Universiti Putra Malaysia, Serdang, Malaysia; ^2^Centre for Community Health Studies, Faculty of Health Sciences, Universiti Kebangsaan Malaysia, Kuala Lumpur, Malaysia; ^3^Faculty of Health and Medical Sciences, University of Adelaide, Adelaide, SA, Australia; ^4^Australian Research Council Centre of Excellence for Nanoscale BioPhotonics, University of Adelaide, Adelaide, SA, Australia

**Keywords:** zerumbone, neuropathic pain, α_2A_-adrenoceptor, TRPV1, NMDA NR2B, allodynia and hyperalgesia

## Abstract

Zerumbone has shown great potential in various pathophysiological models of diseases, particularly in neuropathic pain conditions. Further understanding the mechanisms of action is important to develop zerumbone as a potential anti-nociceptive agent. Numerous receptors and pathways function to inhibit and modulate transmission of pain signals. Previously, we demonstrated involvement of the serotonergic system in zerumbone’s anti-neuropathic effects. The present study was conducted to determine zerumbone’s modulatory potential involving noradrenergic, transient receptor potential vanilloid type 1 (TRPV1) and *N*-methyl-D-aspartate (NMDA) receptors in chronic constriction injury (CCI)-induced *in vitro* and lipopolysaccharide (LPS)-induced SH-SY5Y *in vitro* neuroinflammatory models. von Frey filament and Hargreaves plantar tests were used to assess allodynia and hyperalgesia in the chronic constriction injury-induced neuropathic pain mouse model. Involvement of specific adrenoceptors were investigated using antagonists— prazosin (α_1_-adrenoceptor antagonist), idazoxan (α_2_-adrenoceptor antagonist), metoprolol (β_1_-adrenoceptor antagonist), ICI 118,551 (β_2_-adrenoceptor antagonist), and SR 59230 A (β_3_-adrenoceptor antagonist), co-administered with zerumbone (10 mg/kg). Involvement of excitatory receptors; TRPV and NMDA were conducted using antagonists capsazepine (TRPV1 antagonist) and memantine (NMDA antagonist). Western blot was conducted to investigate the effect of zerumbone on the expression of α_2A_-adrenoceptor, TRPV1 and NMDA NR2B receptors in CCI-induced whole brain samples of mice as well as in LPS-induced SH-SY5Y neuroblastoma cells. Pre-treatment with α_1_- and α_2_-adrenoceptor antagonists significantly attenuated both anti-allodynic and anti-hyperalgesic effects of zerumbone. For β-adrenoceptors, only β_2_-adrenoceptor antagonist significantly reversed the anti-allodynic and anti-hyperalgesic effects of zerumbone. β_1_-adrenoceptor antagonist only reversed the anti-allodynic effect of zerumbone. The anti-allodynic and anti-hyperalgesic effects of zerumbone were both absent when TRPV1 and NMDA receptors were antagonized in both nociceptive assays. Zerumbone treatment markedly decreased the expression of α_2A_-adrenoceptor, while an up-regulation was observed of NMDA NR2B receptors. Expression of TRPV1 receptors however did not significantly change. The *in vitro* study, representing a peripheral model, demonstrated the reduction of both NMDA NR2B and TRPV1 receptors while significantly increasing α_2A_-adrenoceptor expression in contrast to the brain samples. Our current findings suggest that the α_1_-, α_2_-, β_1_- and β_2_-adrenoceptors, TRPV1 and NMDA NR2B are essential for the anti-allodynic and antihyperalgesic effects of zerumbone. Alternatively, we demonstrated the plasticity of these receptors through their response to zerumbone’s administration.

## Introduction

*Zingiber zerumbet* (*Z. zerumbet*) Smith is a wild ginger plant species that has long been used as traditional medicine in Southeast Asia. From rhizomes of *Z. zerumbet*, the main bioactive compound zerumbone has been isolated. Zerumbone has been shown to possess anti-inflammatory (Chien et al., 2008; Sulaiman et al., 2010a), antinociceptive (Sulaiman et al., 2010b), chemopreventive (Murakami et al., 1999), antimicrobial, and anti-oxidative properties (Habsah et al., 2000). Most importantly, we have reported the anti-allodynic and anti-hyperalgesic properties of zerumbone in a neuropathic pain mouse model (Zulazmi et al., 2015; Chia et al., 2016; Zulazmi et al., 2017).

The prevalence of neuropathic pain in the society is unfortunately increasing at a worrying rate. A pain condition due to lesions or diseases that affect the somatosensory nervous system give rise to neuropathic pain (Merskey, 1986). This debilitating chronic pain condition is common to those who suffer from diabetes, tumor nerve compression, viruses (HIV, varicella zoster virus), central nervous system disorders (multiple sclerosis, stroke), and surgical procedures (Baron and Tolle, 2008; Jensen et al., 2009).

The descending pain pathway plays an important role in modulating nociceptive signals, where bidirectional facilitatory or inhibitory control of nociception occurs. The periaqueductal gray (PAG) and rostroventromedial medulla (RVM) have been established as brain structures that provide the most influence on the descending pain pathway (Basbaum and Fields, 1978; Gebhart, 2004; Tracey and Mantyh, 2007). The monoaminergic system mainly utilizes serotonin and noradrenaline neurotransmitters in modulating nociception. These monoamines will act upon their respective subtypes to activate either the descending inhibitory or facilitatory pain pathway (Bannister and Dickenson, 2016).

As we have already shown the involvement of serotonergic system in the anti-neuropathic properties of zerumbone (Chia et al., 2016), this study will further explore the noradrenergic receptors of the monoaminergic system. Projections of noradrenergic neurons to the spinal cord arise from the pontine nuclei, mainly the A5, A6 (locus coeruleus), and A7 (Kölliker-Füse). The PAG and RVM brain structures communicate with these regions to modulate nociceptive transmission (Holden and Proudfit, 1998; Bajic and Proudfit, 1999; Pertovaara, 2006; Bruinstroop et al., 2012). The feedback mechanism of the noradrenergic system in terms of nociceptive modulation occurs following stimulation of sympathetic postganglionic axons, inducing release of noradrenaline neurotransmitters. The neurotransmitter released will then act upon adrenergic receptors to activate downstream effector molecules to inhibit nociceptive transmission (Pertovaara, 2013).

Apart from the descending modulatory controls, other receptors also play a role in inhibiting nociceptive signals. Excitatory receptor; transient receptor potential vanilloid 1 (TRPV1) and *N*-methyl-D-aspartate (NMDA) receptors are known to be involved in transmission of nociception. This is due to their localization on nociceptive neurons and pathophysiological changes in relation to their relative neurotransmitters, altering the activation threshold of action potential (Baron, 2006; Yogeeswari et al., 2009). Targeting of these excitatory receptors through agents that antagonize or agonize have shown promising results. Capsaicin cream, for example, is a TRPV agonist and is clinically used for chronic pain (Anand and Bley, 2011).

Multiple pathways and receptors in our body’s physiological system intertwine to modulate pain signaling pathways. The underlying mechanism of zerumbone’s anti-allodynic and antihyperalgesic effects should be investigated to further potentiate its effectiveness as an analgesic. Therefore, the main objectives of this study were to (1) determine the involvement of the noradrenergic, TRPV and NMDA receptors in the anti-allodynic and antihyperalgesic effects of zerumbone and (2) observe the change in α_2A_-adrenoceptor, TRPV1 and NMDA NR2B receptors expression in the brain regions following zerumbone treatment in neuropathic pain conditions as well as complementing our findings with the *in vitro* LPS-induced SH-SY5Y neuroblastoma neuroinflammation model for peripheral involvement.

## Materials and Methods

### Experimental Animals

Male ICR mice (6–8 weeks, 25–35 g) were used in this study. All mice were housed under a 12 h light/dark cycle at 24 ± 1°C with unlimited access to food and water. Handling of animals and experiments were conducted according to the Ethical Guidelines for Investigation of Experimental Pain in Conscious Animals (Zimmermann, 1983) by the International Association for the Study of Pain (IASP). This study has been approved by the Institutional Animal Care and Use Committee (IACUC) UPM (Ref: UPM/IACUC/AUP- R060/2013).

### Chronic Constriction Injury

The surgery to induce neuropathic pain was adapted from (Bennett and Xie, 1988) with some modifications (Gopalsamy et al., 2019). Briefly, mice were anaesthetized with tribromoethanol (250 mg/kg, i.p.). After shaving the fur on the left thigh region, the sciatic nerve was exposed after an incision was made through the biceps femoris. One loose ligature was placed using a 4-0 braided silk suture until a slight twitch of the left limb was observed. Same surgical procedures were conducted in mice from the sham group, except without ligation of the sciatic nerve. Mice were allowed to recover and behavioral tests were conducted on the 14^th^ day after CCI.

### Zerumbone

Compound extraction and isolation were conducted as previously reported (Chia et al., 2016). Zerumbone was dissolved in dimethylsulfoxide (DMSO), Tween 20 and normal saline (0.99% NaCl) in a ratio of 5:5:90 (v/v). The final concentration of DMSO did not exceed 5% of the total volume and caused no detectable effect on its own. Zerumbone was administered at 10 mg/kg through the intraperitoneal route based on our previous studies (Zulazmi et al., 2015; Chia et al., 2016; Zulazmi et al., 2017). The dosage of zerumbone (10 mg/kg) was chosen based on previous studies published by our colleagues Zulazmi et al. (2015), where they found zerumbone at 10 mg/kg was sufficient to provide anti-allodynic and antihyperalgesic properties in the CCI-induced neuropathic pain mice model. **Figures S1** and **S2** are included as supplementary to provide clarity. In addition, the ED_50_ of zerumbone in a similar neuropathic pain mice model was reported to be 10 mg/kg (Gopalsamy et al., 2017). For the *in vitro* assays, zerumbone was dissolved in phosphate buffered saline (PBS) at 0.25 mg/ml as stock solution.

### Behavioral Tests

#### von Frey Filament Test

Mechanical allodynia was evaluated using the Electronic von Frey Aesthesiometer (IITC, Woodland Hills, CA, USA), adapted from methods by Chaplan et al. (1994). Mice were individually placed in the set-up of clear Plexiglass boxes placed on a wire-mesh platform. The automatic thin steel von Frey filament was positioned under the midplantar surface of the hindpaw. A gradual increase in force was applied until withdrawal of the paw was observed, measuring the maximum force of a mechanical stimulus to elicit a response. Withdrawal thresholds of force greater than 4.5 g was the cut-off point to avoid paw damage.

#### Hargreaves Plantar Test

Thermal hyperalgesia was evaluated using the thermal plantar apparatus (Ugo-basile, 37370, Verase, Italy), adapted from methods by Hargreaves et al. (1988). Mice were individually placed in the set-up clear Plexiglass boxes placed on a glass platform. The radiant heat source was positioned under the midplantar surface of the hindpaw, measuring the withdrawal latency for the mice to lift its paw. Cut-off point to avoid tissue damage was set at 20 s.

### *In Vivo* Analysis of the Mechanisms of Action of Zerumbone

#### Involvement of Noradrenergic System

To firstly investigate the involvement of noradrenergic receptors, non-specific noradrenaline receptor antagonists were used; phentolamine (non-selective α-adrenoceptor antagonist, 5 mg/kg) and propranolol (non-selective β-adrenoceptor antagonist, 5 mg/kg).

Following confirmation of the involvement of α-adrenoceptors, further investigation into the specific noradrenergic receptor subtypes was conducted using selective α-adrenoceptor antagonists; prazosin (α_1_-adrenoceptor antagonist, 10 mg/kg), idazoxan (α_2_-adrenoceptor antagonist, 2 mg/kg). Specific β-adrenoceptor antagonists metoprolol (β_1_-adrenoceptor antagonist, 1 mg/kg), ICI 118,551 (β_2_-adrenoceptor antagonist, 2 mg/kg), and SR 59230 A (β_3_-adrenoceptor antagonist, 2.5 mg/kg) were used following confirmation of the involvement of β-adrenoceptors.

Vehicle or zerumbone (10 mg/kg) were administered 30 min following antagonists’ administration. Following 30 min after last respective treatments, behavioral tests were conducted.

Phentolamine, propranolol, metoprolol, and SR 59230 A were dissolved in 0.9% NaCl, ICI 118,551 was dissolved in 5% DMSO, 95% normal saline (0.9% NaCl) and idazoxan was dissolved in 10% DMSO and 90% normal saline (0.9% NaCl). Phentolamine, propranolol, ICI 118, 551, and SR 59230 A were administered in a volume of 5 ml/kg while idazoxan and metoprolol were administered in a volume of 10 ml/kg. All injections were intraperitoneal, 30 min prior to zerumbone administration. Dosages were chosen based on previous literature (Yalcin et al., 2009a; Yalcin et al., 2009b; Zhao et al., 2012).

#### Involvement of Excitatory Receptors

To assess the possible involvement of excitatory receptors— TRPV1 and NMDA, in the anti-allodynic and antihyperalgesic effects of zerumbone, mice were pre-administered with antagonists prior to zerumbone. Antagonists used were capsazepine (TRPV1 receptor antagonist, 10 mg/kg) and memantine (NMDA receptor antagonist, 10 mg/kg).

Vehicle or zerumbone (10 mg/kg) were administered 30 min following antagonists’ administration. Following 30 min after last respective treatments, behavioral tests were conducted.

Capsazepine and memantine were dissolved in 0.9% NaCl and were administered intraperitoneally, in a volume of 10 ml/kg. Dosages were chosen based on previous studies (Eisenberg et al., 1995; Costa et al., 2008).

#### Western Blot Analysis

Protein analyses were conducted to evaluate the changes in expression level of α_2A_-adrenergic, TRPV1 and NMDA NR2B receptors following neuropathic pain induction and zerumbone treatment. Selection of receptor subtypes were based on behavioral test results and the significant roles played by the receptors in neuropathic pain conditions.

Following behavioral tests, whole brain tissue samples were collected from the experimental animals. Tissue samples were homogenized in cold RIPA lysis buffer with protease inhibitors and the supernatants collected after centrifugation (6,000 g 30 min, 4°C) stored at -20°C until further usage. Sample supernatants (80 μg) were resolved on 8–12% sodium dextran sulfate-polyacrylamide gels, followed by protein transfer to a polyvinylidene fluoride (PVDF) membrane (Pall Life Sciences, Port Washington, NY, USA). The blots were then blocked with 5% Bovine Serum Albumin (BSA) in TBST (Tris Buffer Saline with 0.1% Tween 20) for 1 h. After blocking, blots were incubated overnight with anti- α_2A_ adrenergic receptor (1:500, PA1-048, Thermo Fisher Scientific, USA), anti-VR1 (1:1,000, ab31895, Abcam, USA), or anti-NR2B (1:1,000, ab65783, Abcam, USA) primary antibodies. Blots were then incubated for 1 h with horseradish peroxidase (HRP)-conjugated secondary antibodies (1:5,000, ab97051, Abcam, USA) following sufficient washing with TBST. Blots were washed four times, 20 min each time, with TBST prior to detection using enhanced chemiluminescent (ECL) detection system (Perkin Elmer, USA). Protein bands were analyzed and quantified using ImageJ processing software (National Institutes of Health). Blots were stripped and re-probed with HRP-conjugated anti-β-actin (1:5,000, ab20272, Abcam, USA) for one hour. Bands corresponding to β-actin were normalized to protein bands of samples.

### *In Vitro* Analysis of the Mechanisms of Action of Zerumbone

#### Cell Culture

Dulbecco’s Modified Essential Medium/Ham’s Nutrient Mixture (DMEM:F12), Penicillin-Streptomycin solution and 2.5g/l-Trypsin/1mmol/l-EDTA Solution were purchased from Nacalai Tesque (Tokyo, Japan). Fetal bovine serum (FBS) and non-essential amino acids (NEAA) was purchased from Gibco-BRL (Grand Island, NY). Lipopolysaccharide (LPS) from *Escherichia coli* O55:B5 was purchased from Merck (Darmstadt, Germany).

SH-SY5Y neuroblastoma cell line were purchased from ATCC (ATCC^®^ CRL-2266^™^). The cells were initially grown in Dulbecco’s Modified Essential Medium (DMEM:F12) which contains 4.5 g/l glucose with 2mM of L-glutamine and sodium pyruvate, supplemented with 15% FBS, 1% of Penicillin-Streptomycin mixed solution and 1% NEAA at 37°C with 5% carbon dioxide (CO_2_). Then, the cells were induced with 10 μM of all-*trans* retinoic acid for 5 days in a differentiation media (DMEM:F12, supplemented with 2.5% fetal bovine serum (FBS) and 1% of Penicillin-Streptomycin mixed solution) (Forster et al., 2016; Izham et al., 2018).

#### LPS-Induction and Treatment Groups

Following differentiation, the cells were induced with 1 μg/ml of LPS for 12 h at 37°C with 5% CO_2_ to induce neuronal sensitization (Das et al., 2012). After LPS induction, 8 μg/ml zerumbone, 16 μg/ml amitriptyline as a positive control and vehicle (PBS) were added to the LPS-induced cell culture and incubated for 24 h at 37°C with 5% CO_2_. The whole culture media was not removed and the amount of treatment required were calculated respectively.

#### Western Blot Analysis of α_2A_-Adrenergic, TRPV1 and NMDA NR2B Receptors

In order to extract protein from the cell culture, ice-cold PBS was added to rinse the cell culture following 24 h of treatment. Then, 200 μl of RIPA lysis buffer (with protease inhibitor) was added and the cells were scrapped using the cell scrapper. The sample was then centrifuged at 10,000 rpm for 10 min at 4°C. The supernatant was collected for protein quantification and sodium dodecyl sulfate polyacrylamide gel electrophoresis (SDS-PAGE). The protein concentration was determined by using BCA protein assay (Pierce^™^ BCA Protein Assay Kit). 10 μg of protein sample was prepared by mixing the protein sample and the sample loading buffer (1:1). The protein was separated through SDS-PAGE at 120V for 2 h. Then, the protein was transferred to Polyvinylidene fluoride or polyvinylidene difluoride (PVDF) membrane at 0.35A for 2 h in ice. After transfer, the blot was blocked with 5% skimmed milk in TBST [mixture of tris-buffered saline (TBS) and Tween-20] for 1 h. Then, the blot was incubated overnight at 4°C with polyclonal antibody against β-actin (1: 5,000, #12620, Cell Signalling Technology, Danvers, MA, USA), with monoclonal antibody against GluN2B (NMDA receptor) (1:1,000, #4212, Cell Signalling Technology, Danvers, MA, USA), with polyclonal antibody against Alpha-_2A_ (α_2A_) adrenoceptor (1:1,000, ab85570, Abcam, Cambridge, MA, USA) and with polyclonal antibody against TRPV1 (1:500, bs-1931R, Bioss, MA, USA). After primary antibody incubation, the blots were incubated in secondary antibody (anti-rabbit IgG HRP-linked, 1:2,000, #7074S, Cell Signalling Technology, Danvers, MA, USA) for 1 h at room temperature with continuous agitation. Following incubation, the blots were developed by using ECL solution (Advansta, USA) and the chemiluminescence were detected by ChemiDoc™ imaging system. The band intensity quantification was carried out through the basis of molecular weight by using NIH ImageJ software.

### Data Analysis

All results are expressed as mean ± standard error of mean (S.E.M.). Parametric values were analyzed by one-way ANOVA followed by Tukey’s *post hoc* test using Graphpad Prism v6.0 software (Graphpad San Diego, CA). P values of less than 0.05 were considered significant.

Full images of blots with ladders are provided as supplementary materials, **Figures S3–S5**. Blots shown in the manuscript are images from full blots as provided in the supplementary materials, **Figures S6–S9**.

## Results

### Involvement of Noradrenergic System in the Anti-Allodynic and Antihyperalgesic Effects of Zerumbone

**Figure 1:**
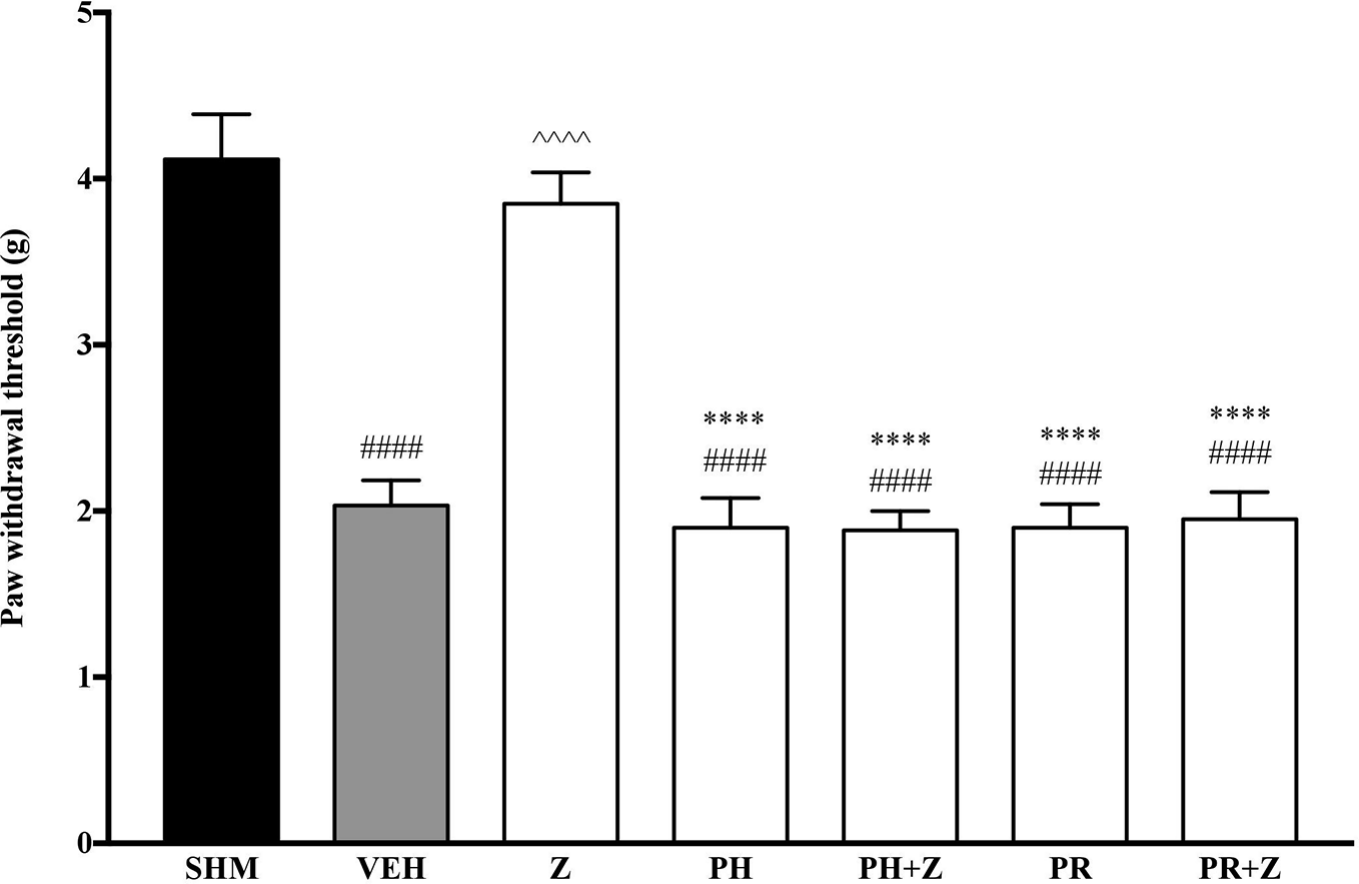
Effect of phentolamine (non-selective α-adrenoceptor antagonist) and propranolol (non-selective β-adrenoceptor antagonist) pre-treatment on zerumbone against mechanical allodynia in CCI-induced neuropathic pain mice. Data are presented as mean ± SEM (n = 6). ^####^*p* < 0.0001 as compared to sham, ^^^^^^*p* < 0.0001 as compared to vehicle and ****p < 0.0001 as compared to zerumbone-treated group. SHM (Sham); VEH (Vehicle, 10 mL/kg i.p.); Z (Zerumbone, 10 mg/kg i.p.); PH (Phentolamine, 5 mg/kg i.p.); PR (Propranolol, 5 mg/kg i.p.).

**Figure 2:**
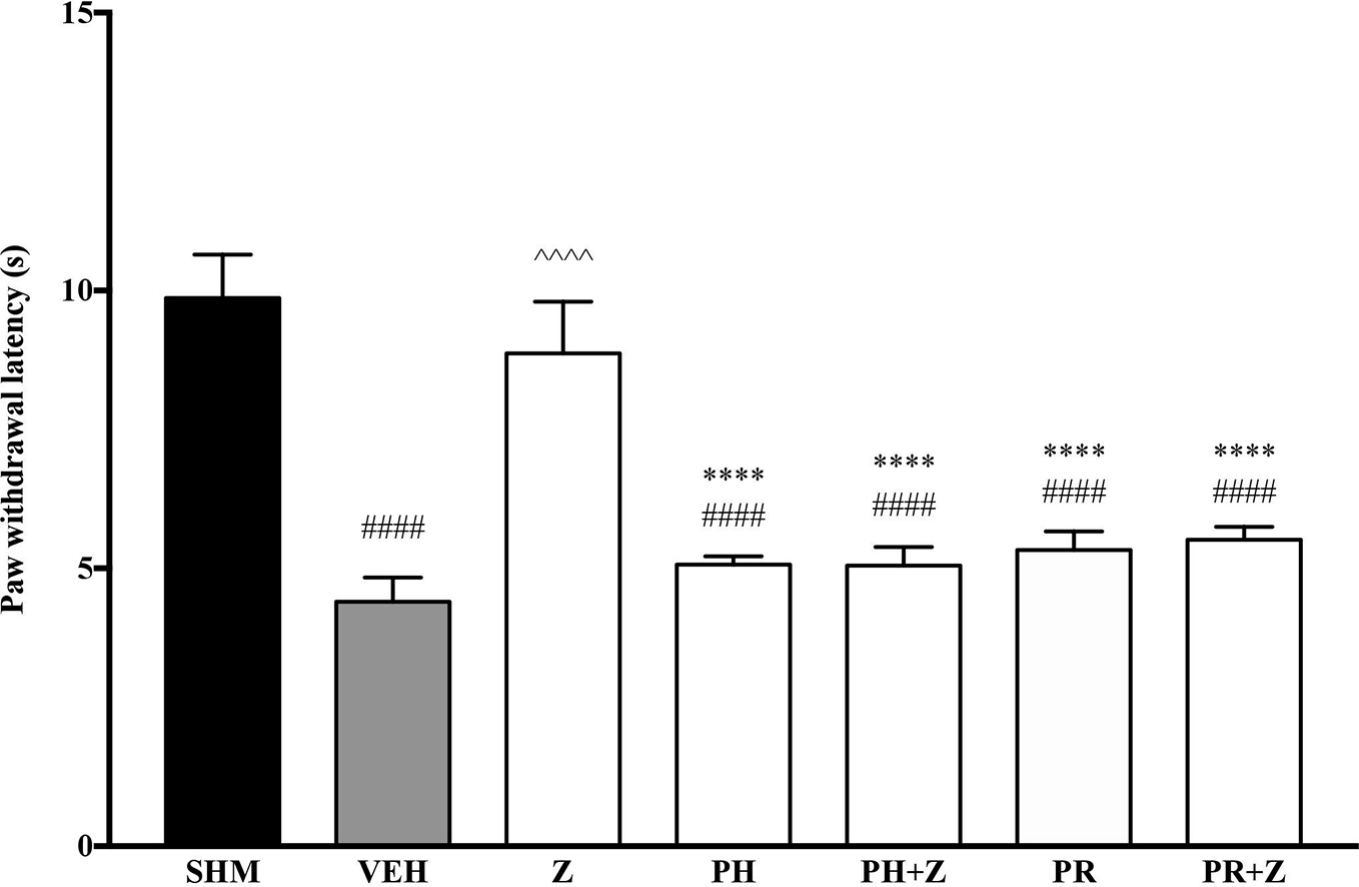
Effect of phentolamine (non-selective α-adrenoceptor antagonist) and propranolol (non-selective β-adrenoceptor antagonist) pre-treatment on zerumbone against thermal hyperalgesia in CCI-induced neuropathic pain mice. Data are presented as mean ± SEM (n = 6) ^####^*p* < 0.0001 as compared to sham, ^^^^*p* < 0.0001 as compared to vehicle and *****p < 0.0001 as compared to zerumbone-treated group. SHM (Sham); VEH (Vehicle, 10 ml/kg i.p.); Z (Zerumbone, 10 mg/kg i.p.); PH (Phentolamine, 5 mg/kg i.p.); PR (Propranolol, 5 mg/kg i.p.).

Before investigating specific adrenoceptors involved in the anti-neuropathic effects of zerumbone, α-adrenoceptors and β-adrenoceptors were non-selectively blocked to determine the involvement of α and β noradrenergic receptors. Phentolamine (5 mg/kg, i,p.) and propranolol (5 mg/kg, i.p.), non-specific α- and β-adrenoceptor antagonists respectively, were pre-administered prior to zerumbone (10 mg/kg, i.p.). Administration of antagonists alone did not significantly affect allodynia and hyperalgesia induced by CCI (**Figure 1**, **Figure 2**). As shown in **Figure 1**, pre-treatment with phentolamine significantly (*p* < 0.0001) abolished the anti-allodynic effect of zerumbone. Similarly, the anti-allodynic effect of zerumbone was also abolished in the presence of propranolol (*p* < 0.0001). In **Figure 2**, similarly pre-treatment with both phentolamine and propranolol attenuated the antihyperalgesic effect of zerumbone.

#### Effects of α-Adrenoceptors Antagonists on Zerumbone-Induced Antineuropathy

**Figure 3:**
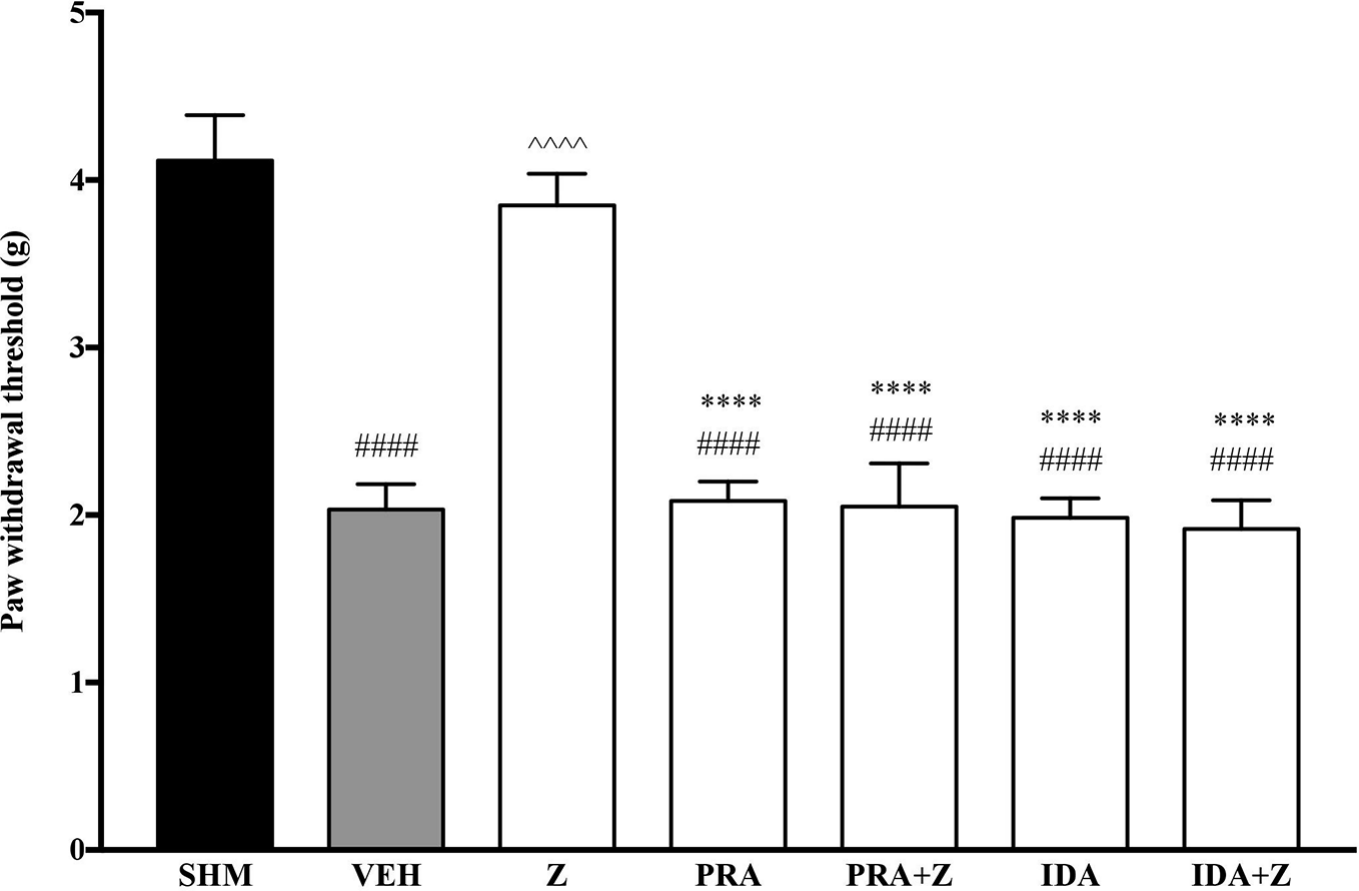
Effect of prazosin (α_1_-adrenoceptor antagonist) and idazoxan (α_2_-adrenoceptor antagonist) pre-treatment on zerumbone against mechanical allodynia in CCI-induced neuropathic pain mice. Data are presented as mean ± SEM (n = 6). ^####^*p* < 0.0001 as compared to sham, ^^^^^^*p* < 0.0001 as compared to vehicle and ****p < 0.0001 as compared to zerumbone-treated group. SHM (Sham); VEH (Vehicle, 10 mL/kg i.p.); Z (Zerumbone, 10 mg/kg i.p.); PRA (Prazosin, 10 mg/kg i.p.); IDA (Idazoxan, 2 mg/kg i.p.).

**Figure 4:**
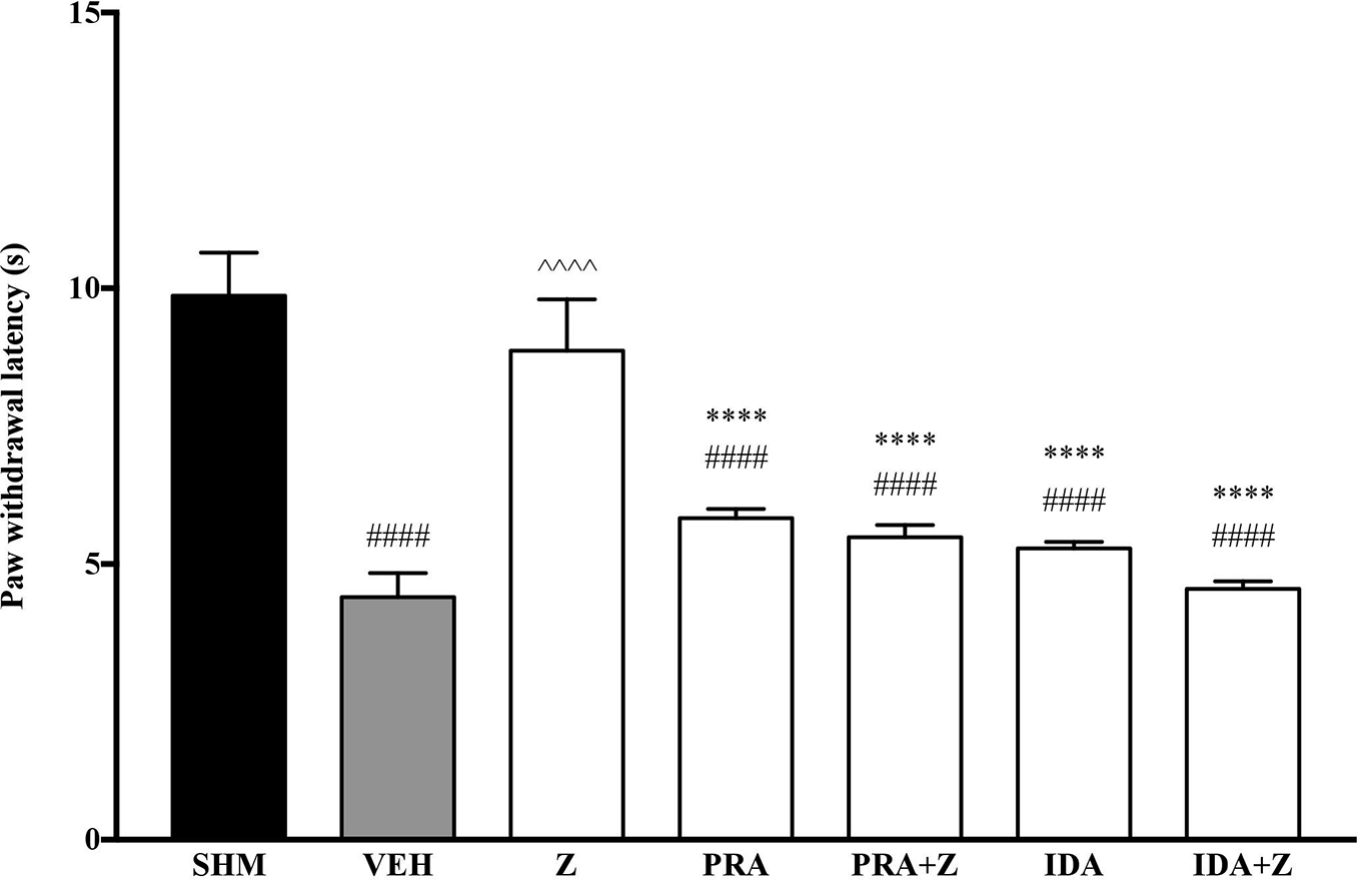
Effect of prazosin (α_1_-adrenoceptor antagonist) and idazoxan (α_2_-adrenoceptor antagonist) pre-treatment on zerumbone against thermal hyperalgesia in CCI-induced neuropathic pain mice. Data are presented as mean ± SEM (n = 6). ^####^*p* < 0.0001 as compared to sham, ^^^^^^*p* < 0.0001 as compared to vehicle and ****p < 0.0001 as compared to zerumbone-treated group. SHM (Sham); VEH (Vehicle, 10 mL/kg i.p.); Z (Zerumbone, 10 mg/kg i.p.); PRA (Prazosin, 10 mg/kg i.p.); IDA (Idazoxan, 2 mg/kg i.p.).

As the non-specific α-adrenoceptor antagonist attenuated the anti-neuropathic effects of zerumbone, further investigation into specific adrenoceptor subtypes were conducted. Specific α-adrenoceptor antagonists to α_1_ and α_2_, prazosin and idazoxan respectively, significantly (*p* < 0.0001) prevented the anti-allodynic effect of zerumbone in the von Frey Filament Test as shown in **Figure 3**. The antihyperalgesic effect of zerumbone was similarly absent (*p* < 0.0001) in the Hargreaves Plantar Test when α_1_- and α_2_-adrenoceptor antagonists were co-administered with zerumbone as shown in **Figure 4**. Administration of antagonists alone did not significantly affect allodynia and hyperalgesia induced by CCI (**Figures 3**, **4**).

### *In Vivo* Analysis of the Mechanisms of Action of Zerumbone

#### Effects of β-Adrenoceptors Antagonists on Zerumbone-Induced Anti-Neuropathy

**Figure 5:**
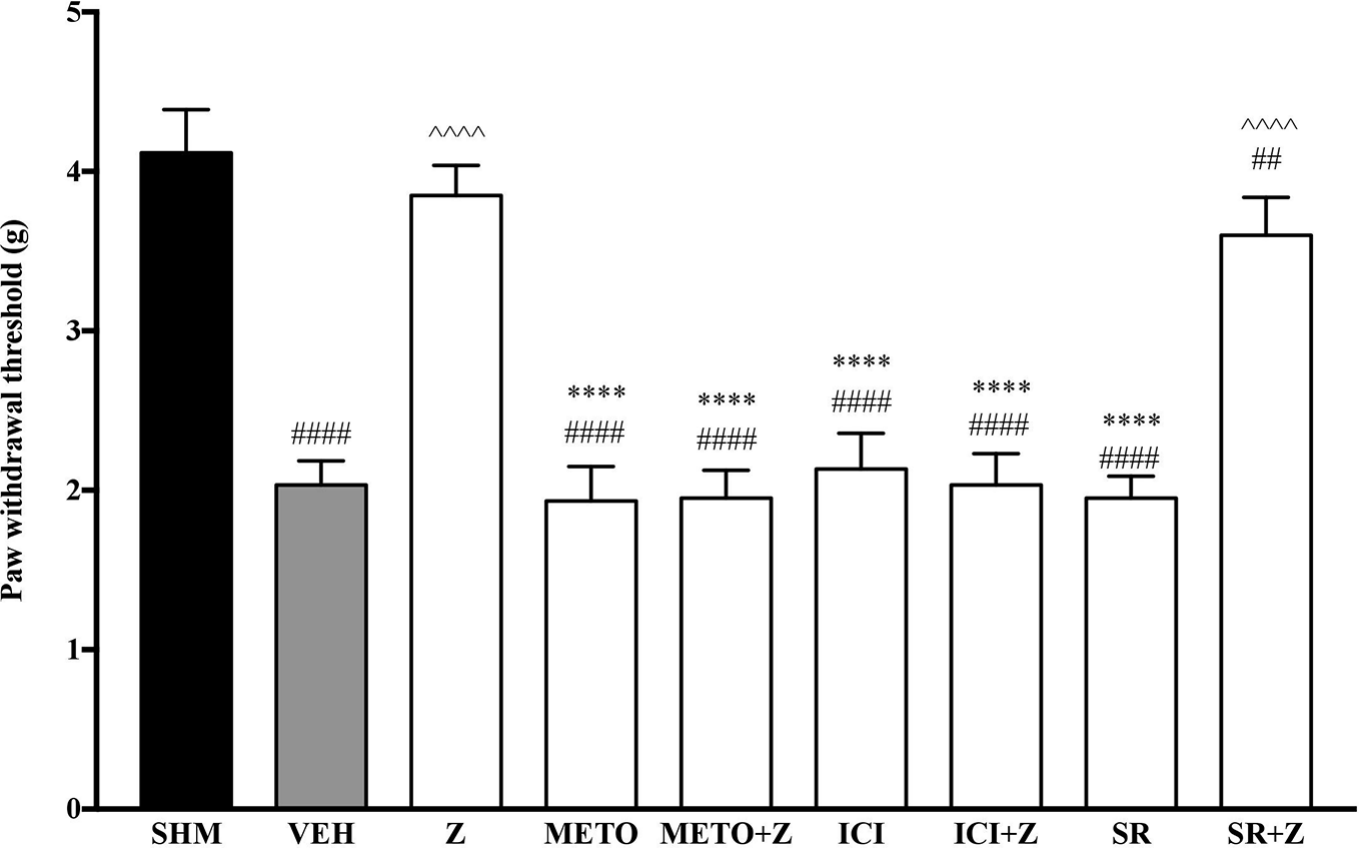
Effect of metoprolol (β_1_-adrenoceptor antagonist), ICI 118, 551 (β_2_-adrenoceptor antagonist) and SR 59230 A (β_3_-adrenoceptor antagonist) pre-treatment on zerumbone against mechanical allodynia in CCI-induced neuropathic pain mice. Data are presented as mean ± SEM (n = 6). ^##^*p* < 0.01, ^####^*p* < 0.0001 as compared to sham, ^^^^^^*p* < 0.0001 as compared to vehicle and ****p < 0.0001 as compared to zerumbone-treated group. SHM (Sham); VEH (Vehicle, 10 mL/kg i.p.); Z (Zerumbone, 10 mg/kg i.p.); METO (Metoprolol, 1 mg/kg i.p.); ICI (ICI 118,551, 2 mg/kg i.p.); SR (SR 59230 A, 2.5 mg/kg i.p.).

**Figure 6:**
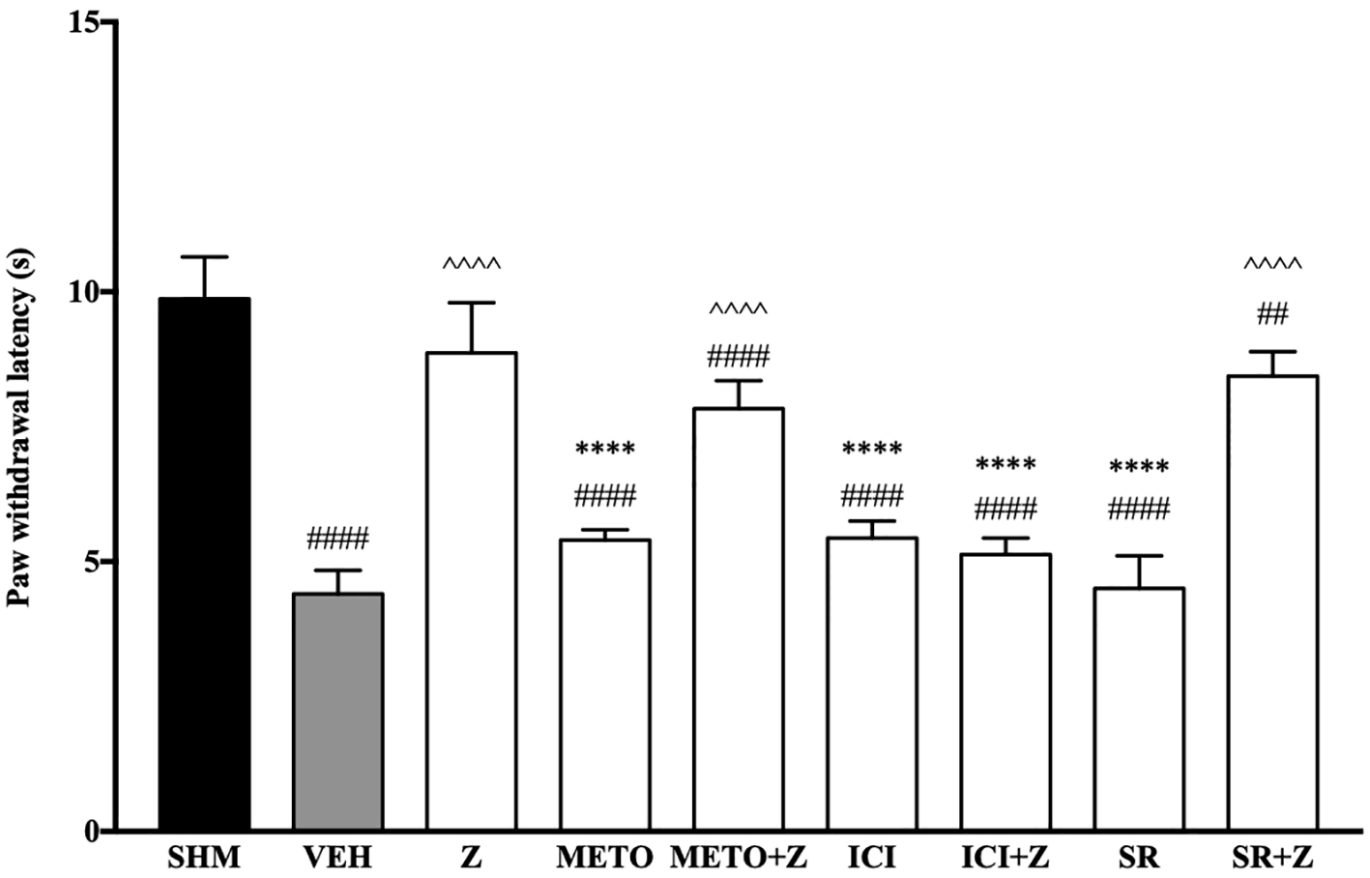
Effect of metoprolol (β_1_-adrenoceptor antagonist), ICI 118, 551 (β_2_-adrenoceptor antagonist) and SR 59230 A (β_3_-adrenoceptor antagonist) pre-treatment on zerumbone against thermal hyperalgesia in CCI-induced neuropathic pain mice. Data are presented as mean ± SEM (n = 6). ^##^*p* < 0.01, ^####^*p* < 0.0001 as compared to sham, ^^^^^^*p* < 0.0001 as compared to vehicle and ****p < 0.0001 as compared to zerumbone-treated group. SHM (Sham); VEH (Vehicle, 10 mL/kg i.p.); Z (Zerumbone, 10 mg/kg i.p.); METO (Metoprolol, 1 mg/kg i.p.); ICI (ICI 118,551, 2 mg/kg i.p.); SR (SR 59230 A, 2.5 mg/kg i.p.).

As pre-administration of the non-selective β-adrenoceptor attenuated the anti-neuropathic effects of zerumbone, specific β-adrenoceptors were then investigated. In **Figure 5**, the anti-allodynic effect of zerumbone was investigated in the presence of β_1_-, β_2_-, and β_3_-adrenoceptor antagonists. Metoprolol and ICI 118, 551, antagonists to β_1_- and β_2_-adrenoceptors respectively, significantly (*p* < 0.0001) attenuated the anti-allodynic effect of zerumbone. However, SR 59230 A, antagonist to β_3_-adrenoceptor, did not reverse the anti-allodynic effect of zerumbone. In **Figure 6**, the antihyperalgesic effect of zerumbone was attenuated (*p* < 0.0001) only in the presence of ICI 118, 551, a β_2_-adrenoceptor antagonist. When metoprolol and SR 59230 A antagonists were pre-administered prior to zerumbone, the withdrawal latency was not significantly different when compared to zerumbone. Administration of antagonists alone did not significantly affect allodynia and hyperalgesia induced by CCI (**Figures 5** and **6**).

#### Effect of Zerumbone on the Expression of α_2A_-Adrenergic Receptor

**Figure 7:**
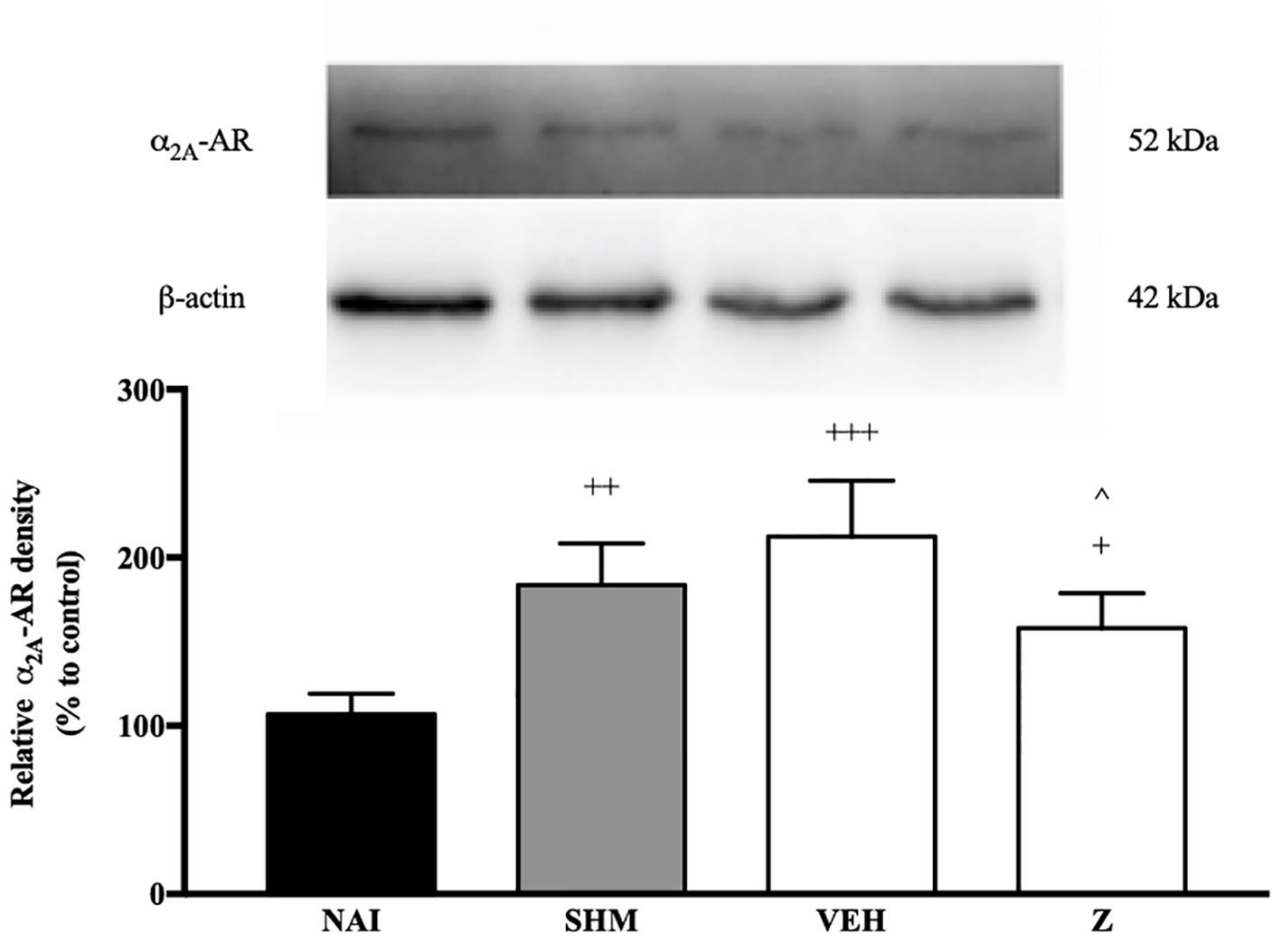
Representative western blots of α_2A_-adrenergic receptor from brain samples of naïve, sham, vehicle and zerumbone-treated groups. Data presented as mean ± SEM (n = 4), which were normalized to β-actin. ^+^*p* < 0.05, ^++^ *p* < 0.01, ^+++^*p* < 0.001 as compared to naïve and ^^^*p* < 0.05 as compared to vehicle group. NAI (Naïve); SHM (Sham); VEH (Vehicle, 10 mL/kg); Z (Zerumbone, 10 mg/kg).

Changes in the expression of α_2A_-adrenoceptor following CCI and zerumbone treatment were assessed using Western blot. Samples from mice brain on Day 14 revealed bands corresponding to α_2A_-AR at ~60 kDa. As shown in **Figure 7**, CCI causes a significant increase in expression of α_2A_-AR as shown between vehicle and naïve groups (*p* < 0.001). In contrast, expression of α_2A_-AR significantly (*p* < 0.05) decreased following zerumbone (10 mg/kg) treatment in comparison to vehicle group.

#### Involvement of TRPV and NMDA Receptors in the Anti-Allodynic and Antihyperalgesic Effects of Zerumbone

**Figure 8:**
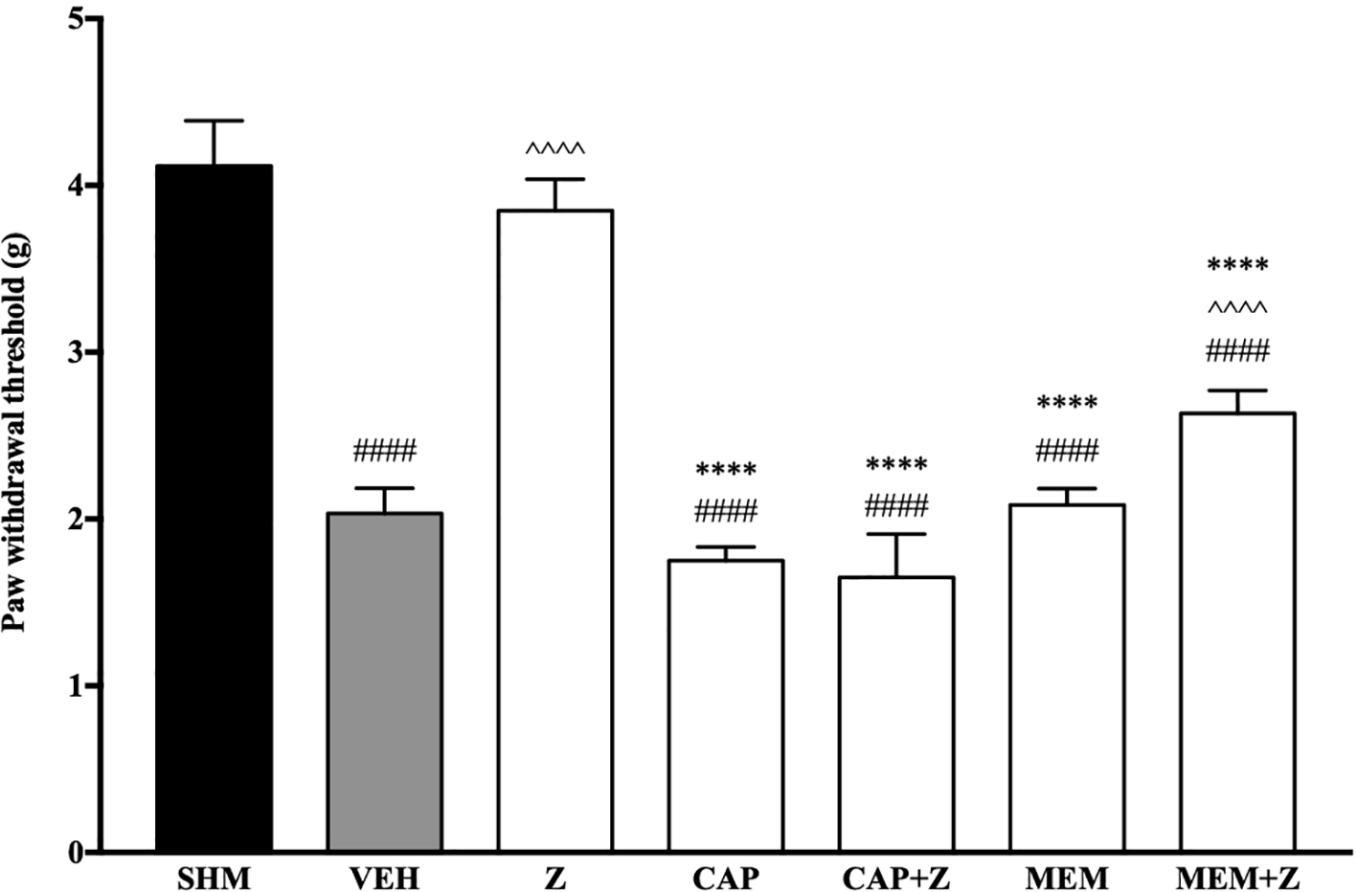
Effect of capsazepine (TRPV1 antagonist) and memantine (NMDA antagonist) pre-treatment on zerumbone against mechanical allodynia in CCI-induced neuropathic pain mice. Data are presented as mean ± SEM (n = 6). ^####^*p* < 0.0001 as compared to sham, ^^^^^^*p* < 0.0001 as compared to vehicle and ^****^p < 0.0001 as compared to zerumbone-treated group. SHM (Sham); VEH (Vehicle, 10 mL/kg i.p.); Z (Zerumbone, 10 mg/kg i.p.); CAP (Capsazepine, 10 mg/kg i.p.); MEM (Memantine, 10 mg/kg i.p.).

**Figure 9:**
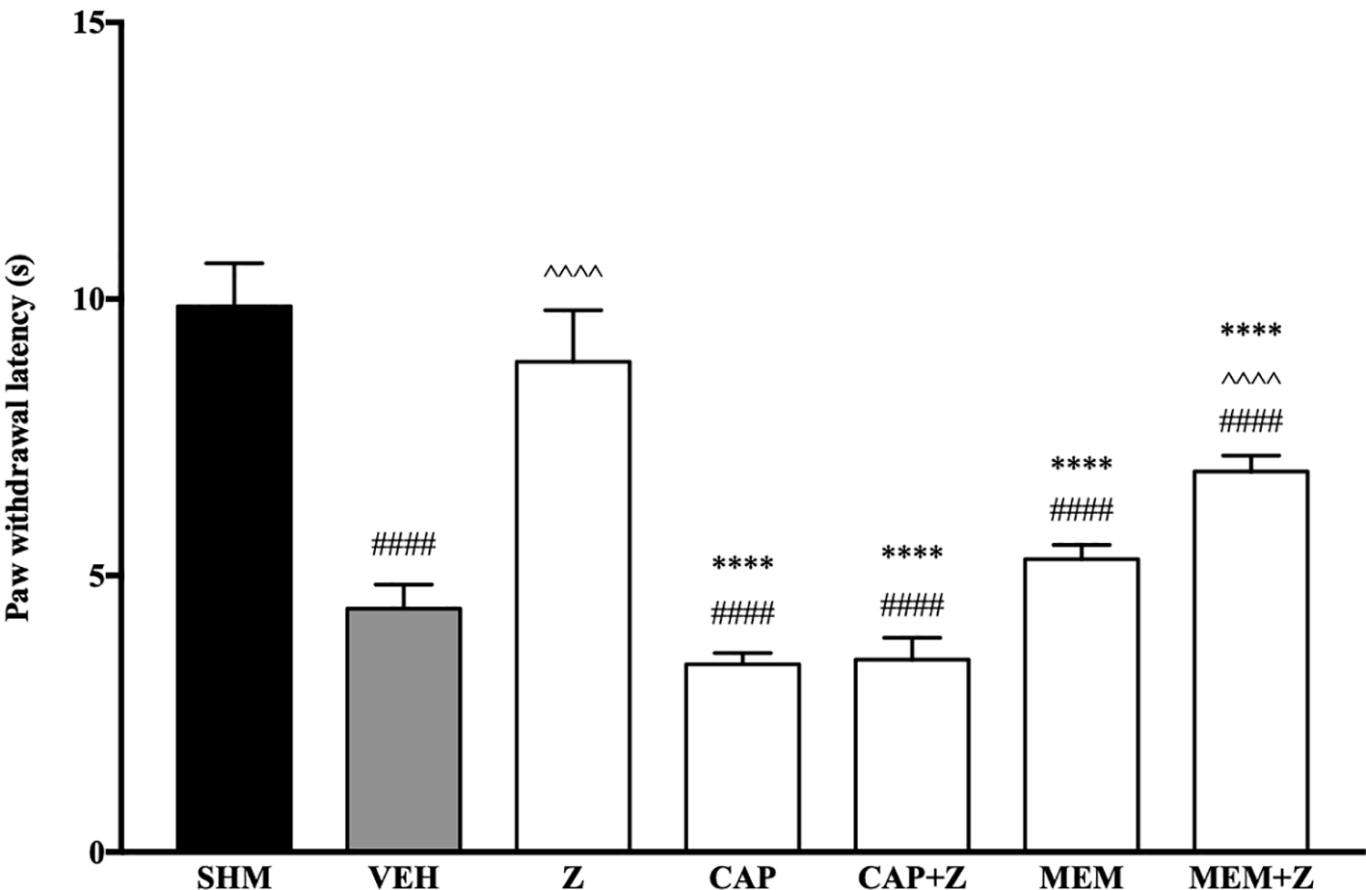
Effect of capsazepine (TRPV1 antagonist) and memantine (NMDA antagonist) pre-treatment on zerumbone against thermal hyperalgesia in CCI-induced neuropathic pain mice. Data are presented as mean ± SEM (n = 6). ^####^*p* < 0.0001 as compared to sham, ^^^^^^*p* < 0.0001 as compared to vehicle and ^****^p < 0.0001 as compared to zerumbone-treated group. SHM (Sham); VEH (Vehicle, 10 mL/kg i.p.); Z (Zerumbone, 10 mg/kg i.p.); CAP (Capsazepine, 10 mg/kg i.p.); MEM (Memantine, 10 mg/kg i.p.).

In **Figure 8**, the anti-allodynic effect of zerumbone was investigated in the presence of TRPV and NMDA receptor antagonists. Pre-treatment with capsazepine and memantine, antagonists to TRPV1 and NMDA respectively, significantly (*p* < 0.0001) attenuated the anti-allodynic effect of zerumbone. Similarly, the antihyperalgesic effect of zerumbone was also absent when antagonists capsazepine and memantine were pre-administered as shown in **Figure 9**. Administration of antagonists on its own did not affect paw withdrawal responses in both behavioral tests (**Figures 8** and **9**).

#### Effect of Zerumbone on the Expression of TRPV1 Receptor

**Figure 10:**
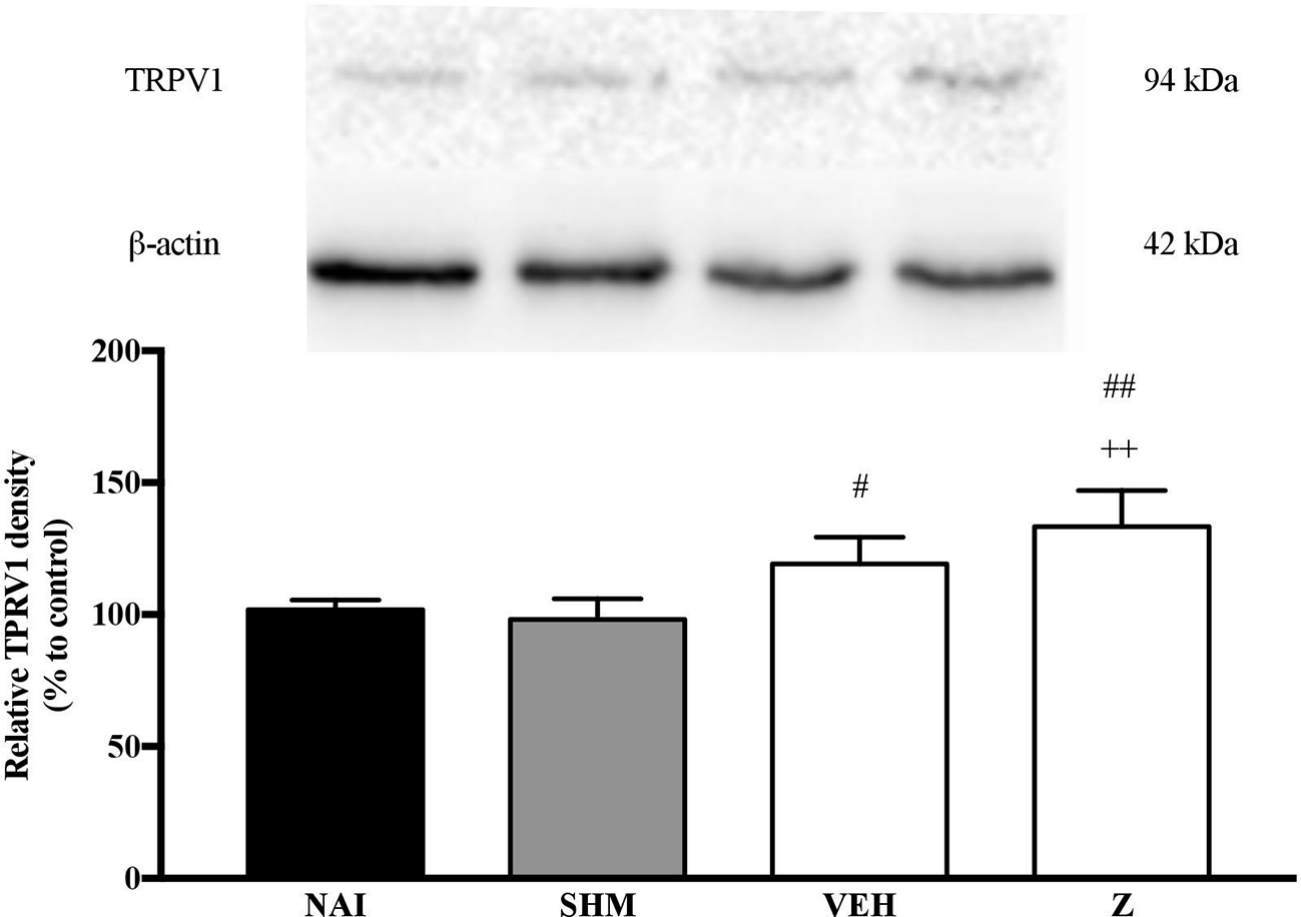
Representative western blots of TRPV1 receptor from brain samples of naïve, sham, vehicle and zerumbone-treated groups. Data presented as mean ± SEM (n = 4), which were normalized to β-actin. ^++^*p* < 0.01 as compared to naïve and ^#^*p* < 0.05, ^##^*p* < 0.01 as compared to sham group. NAI (Naïve); SHM (Sham); VEH (Vehicle, 10 mL/kg); Z (Zerumbone, 10 mg/kg).

Analysis on the expression of TRPV1 receptors were analyzed using brain samples of naïve, sham, vehicle, and zerumbone-treated mice. As shown in **Figure 10**, the bands observed corresponded to the expected molecular weight ~94 kDa. The induction of neuropathic pain caused a significant (*p* < 0.05) up-regulation of TRPV1 receptors, when comparing vehicle against sham group. No significant changes were observed between vehicle and zerumbone-treated groups. However, expression of TRPV1 receptors in zerumbone-treated groups is significantly (*p* < 0.01) higher against naïve and sham groups.

#### Effect of Zerumbone on the Expression of NMDA NR2B Receptor

**Figure 11:**
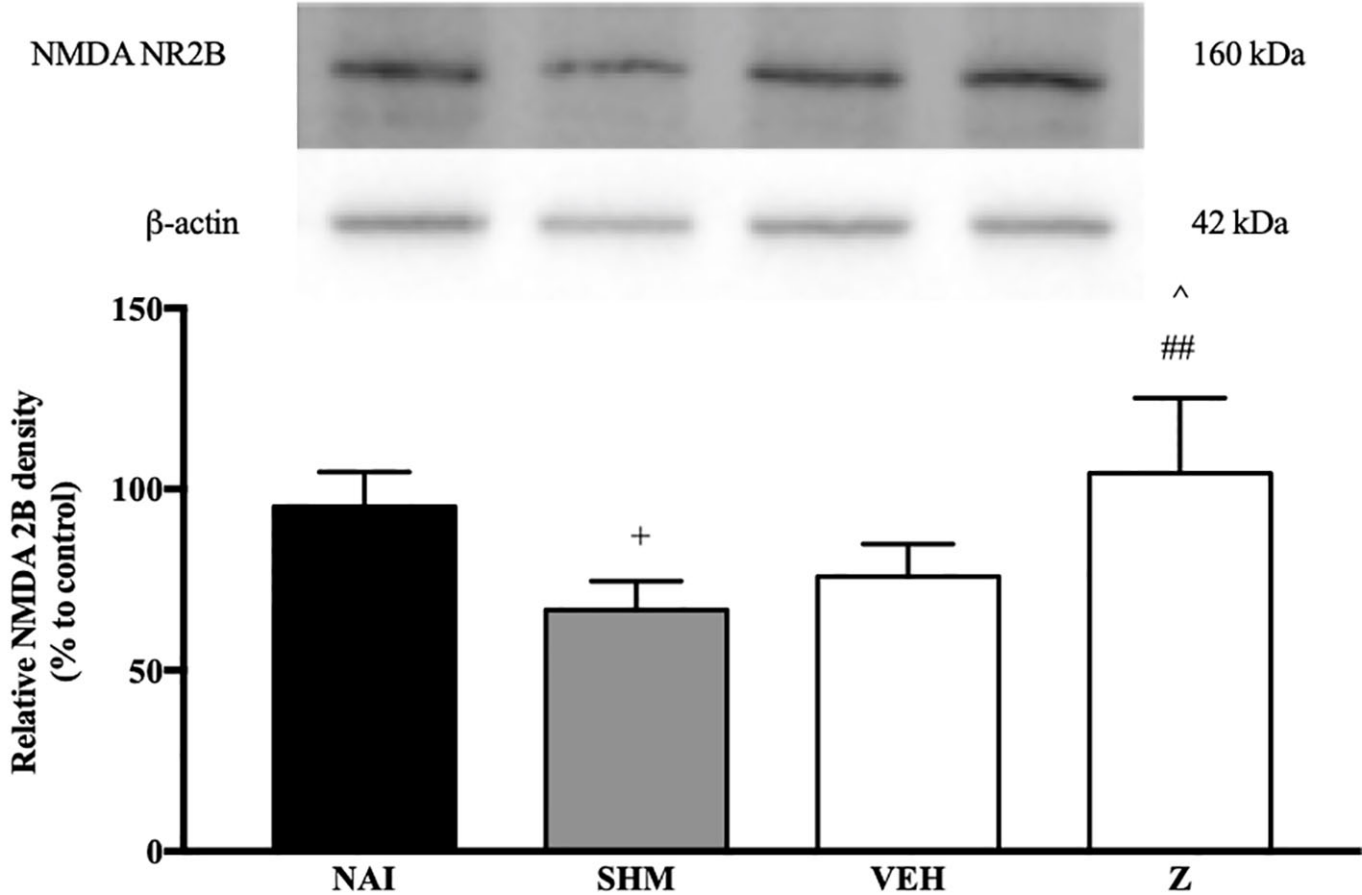
Representative western blots of NMDA NR2B receptor from brain samples of naïve, sham, vehicle and zerumbone-treated groups. Data presented as mean ± SEM (n = 4), which were normalized to β-actin. ^+^*p* < 0.05 as compared to naïve, ^##^*p* < 0.01 as compared to sham group and ^^^*p* < 0.05 as compared to vehicle. NAI (Naïve); SHM (Sham); VEH (Vehicle, 10 mL/kg); Z (Zerumbone, 10 mg/kg).

The changes on NMDA NR2B receptor expression were analyzed following CCI and zerumbone treatment. As shown in **Figure 11**, the bands observed corresponded to the expected molecular weight ~160 kDa. In vehicle group, no significant changes were observed of the NR2B receptor expression in comparison to naïve and sham groups. However, a significant (*p* < 0.05, *p* < 0.01) up-regulation was observed in zerumbone-treated groups, compared against sham and vehicle groups.

### *In Vitro* Analysis of the Mechanisms of Action of Zerumbone

#### Western Blot Analysis of α_2A_-Adrenergic, TRPV1 and NMDA NR2B Receptors

**Figure 12:**
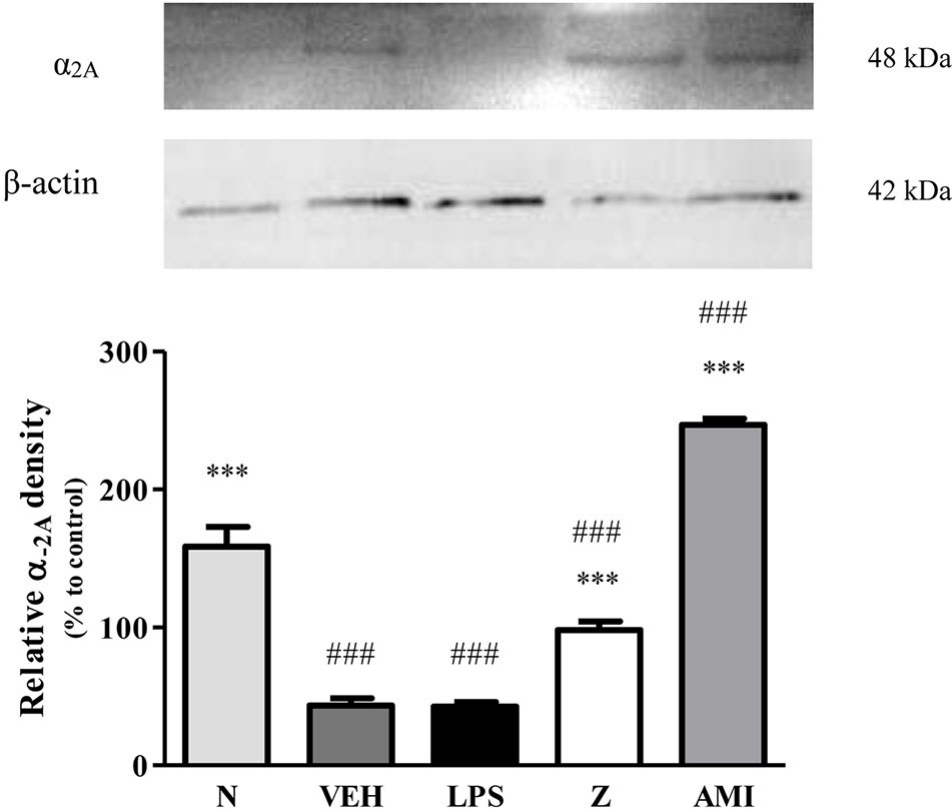
Representative western blots of α_2A_-adrenergic receptor from LPS-induced SH-SY5Y cells samples of normal, vehicle, LPS only, zerumbone, amitriptyline-treated groups. Data presented as mean ± SEM (n = 4), which were normalized to β-actin. ***p < 0.001 as compared to LPS only group and ^###^*p* < 0.001 as compared to normal group. N (Normal); VEH (Vehicle, PBS); Z (Zerumbone, 8 μg/ml); AMI (Amitriptyline, 16 μg/ml).

**Figure 13:**
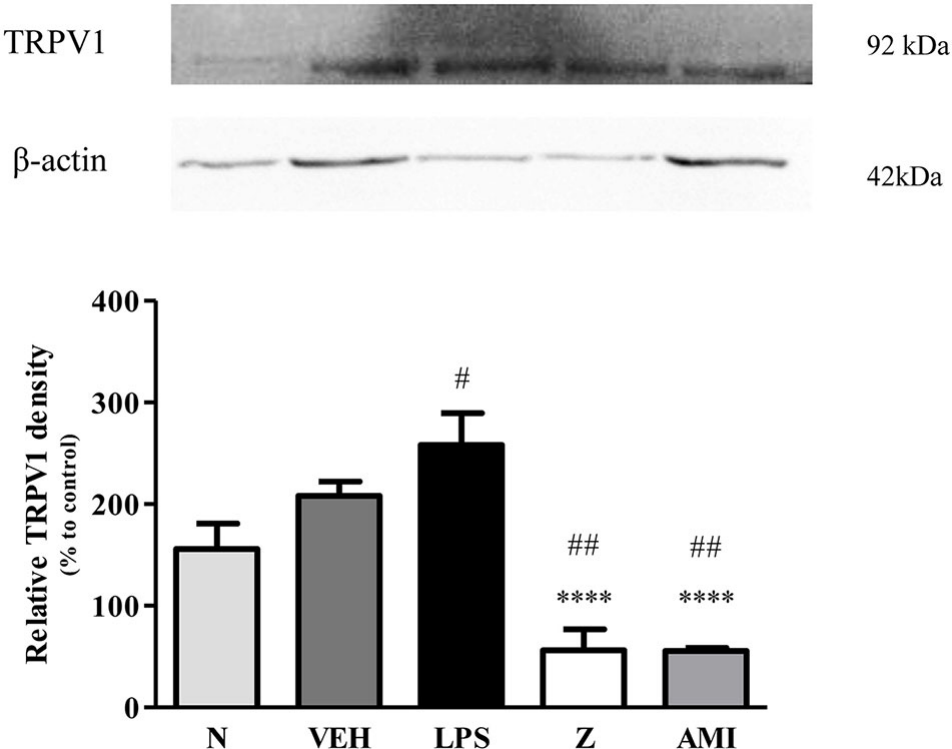
Representative western blots of TRPV1 channel from LPS-induced SH-SY5Y cells samples of normal, vehicle, LPS only, zerumbone, amitriptyline-treated groups. Data presented as mean ± SEM (n = 4), which were normalized to β-actin. ****p < 0.0001 as compared to LPS only group and ^#^*p* < 0.1, ^##^*p* < 0.01 as compared to normal group. N (Normal); VEH (Vehicle, PBS); Z (Zerumbone, 8 μg/ml); AMI (Amitriptyline, 16 μg/ml).

**Figure 14:**
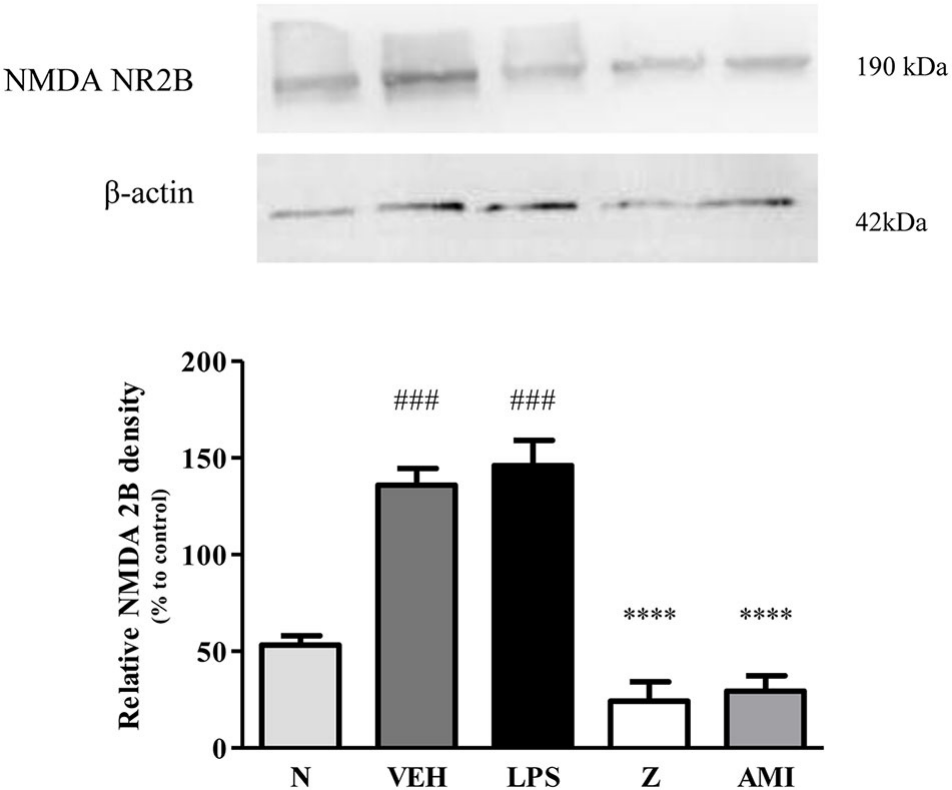
Representative western blots of NMDA NR2B receptor from LPS-induced SH-SY5Y cells samples of normal, vehicle, LPS only, zerumbone, amitriptyline-treated groups. Data presented as mean ± SEM (n = 4), which were normalized to β-actin. ****p < 0.0001 as compared to LPS only group and ^###^*p* < 0.001 as compared to normal group. N (Normal); VEH (Vehicle, PBS); Z (Zerumbone, 8 μg/ml); AMI (Amitriptyline, 16 μg/ml).

Changes in the expression of α_2A_-adrenergic, TRPV1, and NMDA NR2B receptors in the LPS-induced SH-SY5Y neuroblastoma cells were analyzed 24 h after the administration of 8 μg/ml zerumbone, 16 μg/ml amitriptyline, and vehicle. As shown in **Figure 12**, zerumbone administration significantly increased the expression of α_2A_-adrenergic receptors by the SH-SY5Y cells. In contrast, both the TRPV1 and NMDA NR2B receptors were down-regulated following the treatment with zerumbone as shown in **Figures 13** and **14**. Additionally, the *in vitro* findings are in contrast to the receptor’s expression in the *in vivo* brain regions where, α_2A_-adrenergic receptors were down-regulated while the TRPV1 and NMDA NR2B receptors were up-regulated.

## Discussion and Conclusion

We have previously demonstrated the antinociceptive properties of zerumbone in the chronic constriction injury neuropathic pain mice model and its mechanisms through serotoninergic (Chia et al., 2016) and the L-arginine–nitric oxide (Zulazmi et al., 2017) pathways. With this study, we now show that the noradrenergic system and excitatory receptors are crucial to zerumbone’s anti-allodynic and antihyperalgesic properties. Our current study suggests that zerumbone produces its anti-allodynic properties by interacting with α_1_-, α_2_-, β_1_-, and β_2_-adrenergic receptors. Meanwhile, α_1_-, α_2_-, β_2_-adrenergic receptors are responsible for zerumbone’s antihyperalgesic property. Excitatory receptors TRPV and NMDA are involved in both zerumbone-induced anti-allodynia and antihyperalgesia. Therefore, we hypothesize that a synergistic mechanism between noradrenaline, TRPV, and NMDA is utilized by zerumbone to produce its anti-allodynic and antihyperalgesic properties.

The noradrenergic system is part of the descending monoaminergic pain modulation pathway. The serotonergic system is known to exert both inhibitory as well as excitatory effects in pain modulation, whereas the noradrenergic system predominantly has an inhibitory role in pain modulation (Suzuki et al., 2004b). Two main classes of adrenergic receptors (AR) arise from the noradrenergic projections from the locus coeruleus (LC), which are the α and β ARs (Millan, 2002; Hentall et al., 2003). Both α and β ARs and their subtypes are G protein-coupled receptors (GPCR), thus their main action after binding of noradrenaline differs depending on the sub-class of G proteins each receptor couples to (Llorca-Torralba et al., 2016).

Following nerve injury, the central and peripheral nervous system undergoes physiological changes. Possible alterations to descending monoaminergic pathway influences neurotransmitter metabolism and/or number and affinity of receptor uptake sites lead to neuropathic pain (Suzuki et al., 2004a; Rahman et al., 2008; Leong et al., 2011). The induction of neuropathic pain was measured by the endurance of the animals through behavioral tests on their pain threshold. Firstly, we investigated the involvement of noradrenergic receptors by administering non-selective α- and β-adrenoceptor antagonists, phentolamine and propranolol respectively. In the presence of phentolamine and propranolol, both the anti-allodynic and antihyperalgesic properties of zerumbone we attenuated. Thus, further examinations into specific receptors to the noradrenergic system were conducted.

Administration of prazosin, a selective α_1_-AR antagonist prior to zerumbone treatment managed to abolish zerumbone’s anti-neuropathic properties. The α_1_ adrenoceptors are G_q/11_ protein receptors, which are coupled to phospholipase C (PLC) (Bylund et al., 1994). Binding of noradrenaline to α_1_-AR causes increase in intracellular calcium pool as a result of hydrolysis of inositol phosphates, with diacyl glycerol (DAG) and inositol triphosphate (IP_3_) as its products (Millan, 2002). The α_1_-AR has been implicated to facilitate nociception (Millan, 1999; Fuchs et al., 2001; Hord et al., 2001b), and is said to contribute to the development of chronic pain. However, previous studies have reported antinociceptive activity when α_1_-AR agonists were used, possibly acting pre-synaptically on central primary afferent nociceptors (Howe et al., 1983; Kawabata et al., 1994; Hord et al., 2001a). As discussed by Millan (2002), the bidirectional reports on both pro- and anti-nociceptive effects of α_1_-AR could be due to co-localization of α_1_- and α_2_-AR.

As with the α_1_-AR antagonist, the anti-allodynic and antihyperalgesic properties of zerumbone were absent when α_2_-AR antagonist, idazoxan, was administered prior to zerumbone treatment. Unlike α_1_-AR, α_2_-adrenoceptors are coupled to G_i/o_ proteins, which alters membrane polarization through K^+^ and Ca^2+^ channels (Millan, 2002). Activation of α_2_-AR results in intracellular changes whereby cAMP levels are decreased due to inhibition of adenylyl cyclase. The α_2_-AR is the most commonly implicated adrenergic receptor to be responsible in inhibiting pain transmission. Pre-synaptically, the α_2_-AR plays an important inhibitory feedback mechanism in the release of noradrenaline from adrenergic neurons (Gilsbach and Hein, 2008). There are three subtypes to α_2_-AR, which are the 2A, 2B, and 2C receptor subtypes. α_2A_- and α_2C_-AR are widely expressed in the central nervous system (CNS) while the α_2B_-AR can be commonly found in non-neuronal tissues. The α_2A_-AR is the predominant subtype found in the brainstem.

Based on the results obtained in the present study, zerumbone utilizes the α_2_-AR in exhibiting its anti-neuropathic effect. Activation of α_2_-AR causes an increase in neuronal firing activity from the LC and studies have found that activation from a α_2_-AR agonist to decrease noradrenaline (NA) concentration in the prefrontal cortex (PFC) (Svensson et al., 1975; Pudovkina et al., 2001; Jedema et al., 2008). However, previous studies have reported that the inhibitory actions of α_2_-AR to be absent in neuropathic conditions (Xu et al., 2000; Obata et al., 2005; Omiya et al., 2008; Chen et al., 2011). Thus, the antinociceptive activity due to α_2_-AR activation is said to originate from the LC, to compensate in the loss of spinal α_2_-adrenergic receptor activity. Alternatively, Alba-Delgado et al. (2012) has proposed that α_2_-AR desensitization to occur in the LC that enhances the antinociceptive noradrenergic effects in neuropathic pain conditions.

β-adrenoceptors can be further classified into β_1_-, β_2_-, and β_3_-adrenergic receptors (Bylund et al., 1994). All three β-AR are G_s_ proteins, coupled to adenylyl cyclase to increase intracellular secondary messenger cAMP synthesis. These receptors are widely distributed in the CNS (Nicholas et al., 1993). Although most of the focus on the noradrenergic system is on the α_2_ subtype, studies have shown that β-AR are also involved in pain modulation (Brochet et al., 1986; Choucair-Jaafar et al., 2009).

The zerumbone-induced anti-allodynic and antihyperalgesic effects were significantly reversed by administration of ICI 118,551, a β_2_-AR antagonists, but not of SR 59230 A, a β_3_-AR antagonist. Metoprolol, a β_1_-AR antagonist, only attenuated the anti-allodynic effect of zerumbone. β_1_- and β_2_-AR are found in both central and peripheral nervous systems, with the β_1_-AR densely expressed in cerebral cortex, thalamus, and sympathetic ganglia whereas β_2_-AR to localize more in the olfactory bulb, hippocampus, hypothalamus, and spinal cord (Nicholas et al., 1996; Gilsbach and Hein, 2008). As mentioned, the α_2_-adrenergic receptors are the predominant adrenergic receptors to inhibit nociceptive transmission. Thus, not many studies have been conducted on β-adrenergic receptor subtypes. However, studies have shown that β_2_-AR is necessary for antidepressants to exhibit its anti-neuropathic effects (Yalcin et al., 2009a; Yalcin et al., 2009b). It is possible that the activation of downstream proteins due to β_2_-AR facilitates protein kinase A activation by cAMP, which results in enhanced NA release from sympathetic nerves (Boehm and Kubista, 2002; Kubista and Boehm, 2006).

With consideration to our current findings and in line with literature on the more prominent role of α_2_-adrenergic receptors, primarily the α_2A_ subtype, we investigated whether the α_2A_-adrenoceptor is involved in zerumbone’s anti-neuropathic effects. Our findings have shown (**Figure 7**) that chronic constriction injury induces an increase in expression of α_2A_-adrenergic receptors in the brain. Previous studies have also reported similar findings, where nerve lesions causes an up-regulation in α_2A_-adrenergic receptor expression as early as 7 days following injury induction (Alba-Delgado et al., 2013).

In normal conditions, the noradrenergic system primarily functions to inhibit nociceptive transmission. Following nerve injury, the plastic changes that occur shift the inhibitory tone of the noradrenergic system to facilitate nociceptive transmission instead (Brightwell and Taylor, 2009; Kaushal et al., 2016). Therefore, the increase in expression of α_2A_-adrenoceptor following CCI in vehicle-treated group may be due to plastic changes that occur, abolishing the inhibitory tone of the noradrenergic system. In support of this hypothesis, zerumbone treatment suppressed the increased expression of α_2A_-AR as shown in this study. Development of neuropathic pain is the result of cumulative plastic changes that occur throughout the nervous system. Our findings as shown in **Figure 7** indicates that the expression of α_2A_-AR decreases upon zerumbone administration. It is possible that the primary action of zerumbone in attenuating allodynia and hyperalgesia is through suppression of the α_2A_-AR up-regulation.

Therefore, the cumulative action of zerumbone against neuropathic pain might not only utilize the descending noradrenergic pathway, but also through the noradrenergic projections to the other brain sites involved in the pain pathway. In particular, the rostroventromedial medulla and periaqueductal gray brain regions are important in modulating nociceptive signals (Pertovaara, 2006). Moreover, adrenoceptors are also localized on the descending serotonergic pathway. The inhibitory α_2_-AR especially, has been reported to be highly concentrated in serotonergic neurons (Rosin et al., 1993; Guyenet et al., 1994; Millan et al., 2000; Millan, 2002).

Our current findings implicate the involvement of TRPV1 and NMDA NR2B receptors in zerumbone’s anti-allodynic and antihyperalgesic properties. Zerumbone and the essential oil of *Zingiber zerumbet* have been associated with TRPV and glutamatergic (NMDA) system in its mechanistic actions against acute pain (Khalid et al., 2011; Perimal et al., 2011). Due to similarities between acute and chronic pain pathways, zerumbone is therefore implicated to also involve TRPV and NMDA receptors in exerting its anti-neuropathic properties in the CCI model of neuropathic pain.

The families of transient receptor potential (TRP) ion channels are primarily expressed on nociceptive neurons. TRPV1 receptors in particular, were discovered to be involved in nociceptive processing due to capsaicin, an active component from *Capsicum* chili peppers (Caterina et al., 1997). Acidic conditions, high temperatures, and noxious stimuli activate TRPV1 receptors. In the pathogenesis of neuropathic pain, expression of TRPV1 is up-regulated on uninjured A- and C-fibers, as well as injured dorsal root ganglions. These alterations to receptor expression causes an amplification of noxious stimuli resulting in peripheral sensitization (Baron, 2006).

Zerumbone is hypothesized to exert either an antagonist-like effect or desensitize TRPV1 receptors to suppress mechanical allodynia and thermal hyperalgesia. Previous studies have reported that TRPV1 antagonists are able to alleviate nociception. Yamamoto et al. (2008) reported the TRPV1 antagonist, N-(4-Tertiarybutylphenyl)-4-(3-chloropyridin-2-yl) tetrahydropyrazine-1(2H)-carbox-amide (BCTC), suppressed mechanical allodynia in the CCI neuropathic pain model. As a relative comparison to zerumbone, curcumin has also exhibited potent analgesic properties (Sharma et al., 2006; Mittal et al., 2009; Zhao et al., 2014). Curcumin, the active ingredient of turmeric (*Curcuma longa*), with several of its analogues behave as antagonists on TRP channels (Nalli et al., 2017). Similarly, Yeon et al. (2010) observed suppression of TRPV activation by curcumin, thereby exhibiting antihyperalgesic effect.

On the contrary, zerumbone could also act as an agonist to desensitize TRPV receptors. Similar mechanisms can be observed with capsaicin, a TRPV agonist. Many over-the-counter topical creams contain low concentrations of capsaicin, typically used as an analgesic. Analgesia produced from TRPV activation occurs due to the lasting refractory rate. This desensitization phase causes the excitable receptors to be insensitive to any noxious stimuli (Perry et al., 2007). Among the ligands that are able to activate the TRPV1 channels are vanilloids (e.g.: resineferatoxin, capsaicin), protons, endogenous lipids, polyamines, and noxious heat (Caterina et al., 1997; Zygmunt et al., 1999; Ahern et al., 2005; Ahern et al., 2006).

The noradrenergic system and TRPV receptors have shown their interrelation in the pain pathway. Our findings show zerumbone utilizes noradrenergic receptors as well as TRPV receptors in eliciting its anti-neuropathic properties. Recently, Chakraborty et al. (2017) found that noradrenaline released *via* the descending noradrenergic system inhibits pre-synaptic TRPV1 channels. Therefore, it is possible that the descending noradrenergic system enhances the inhibition on TRPV1 channels in the presence of zerumbone to attenuate neuropathic pain symptoms.

Further analysis into expression of TRPV1 receptors on mice in neuropathic pain conditions shows a significant increase. The present findings observed a slight increase in TRPV1 expression in comparison to neuropathic pain mice. An up-regulation in TRPV receptor expression has been reported. On the basis of their known mechanisms, the increase in expression of the excitatory receptor TRPV1 is therefore implicated in the enhanced excitability state of nociceptive transmission. As a result, the peripheral sensitization that occurs soon develops to neuropathic pain (Mannion et al., 1999; Ji et al., 2002).

Expression of TRPV1 in brain is primarily in microglial cells and in discrete amounts at other brain regions such as anterior cingulate cortex (ACC). TRPV1 activation modulates synaptic neurotransmission and indirectly enhances glutamatergic neuronal transmission, heightening nociceptive transmission leading to pathophysiological persistence of pain (Marrone et al., 2017). However, recent advances by Silva et al. (2016) have also provided new evidences in the inhibitory influence of TRPV1 channels in modulating nociceptive transmission. The modulatory role is reportedly controlled by the expression of TRPV1 on the rostral ventromedial medulla (RVM), in contrast to ACC region mentioned earlier. Therefore, the inhibitory role of TRPV1 up-regulation from zerumbone treatment is possibly implicated by its modulatory role expressed by TRPV1 in the RVM.

NMDA is an ionotropic glutamate receptor. NMDA receptors bind to glutamate, the major excitatory neurotransmitter of the central nervous system. Glutamate and its receptors are the major contributors to the development of neuropathic pain through central sensitization (Bennett, 2000). Activation of this class of excitatory receptors causes an influx of Ca^2+^, activation of nitric oxide synthases (NOS) and cyclooxygenase-2 (COX-2). The disproportionate availability of nitric oxide and prostaglandin results in prolonged excitation of neural and glial cells (Fundytus, 2001).

The present study shows that zerumbone partially utilizes NMDA receptors to elicit its anti-allodynic and antihyperalgesic effects. These results conform to previous study on zerumbone in an animal model of acute pain where zerumbone dose-dependently inhibited glutamate-induced nociception (Perimal et al., 2011). Considering the excitatory functioning on NMDA receptors, antagonists to these receptors are now considered as clinical analgesics. Zerumbone is therefore postulated to act as an antagonist on NMDA receptors, thus suppressing calcium ions influx to dampen nociceptive transmission. The action of zerumbone on glutamatergic transmission may occur either peripherally or centrally.

Several populations of glutamatergic receptors, including NMDA, are known to localize on noradrenergic terminals. Presynaptic activation these NMDA receptors regulates the release of NA (Forray et al., 2000; Luccini et al., 2007). Increased availability of NA thus enhances the inhibitory tone of the noradrenergic system. A *vice versa* mechanisms have also been reported, where activation of the locus coeruleus noradrenergic system causes a down-regulation of NMDA receptors (Roh et al., 2008; Kang et al., 2012). Therefore, our current findings imply that the mechanistic action of zerumbone is not specific to a single pathway, but rather a summative effect through various inhibitory and excitatory receptors.

The NMDA NR2B subunit was chosen to further analyze the effects of zerumbone. Activation of NR2B subunit contributes to the excitatory role of NMDA receptors as it induces c-Jun N-terminal kinase (JNK) activation and enhances astrocytic-neuronal signaling (Kato et al., 2006). The NR2B subunit of NMDA receptors are regionally distributed, however it is primarily expressed superficially at the dorsal horn and is highly associated with nociceptive transmission (Laurie et al., 1997). In the present study, it was found that expression of NMDA NR2B in CCI mice did not significantly increase in comparison to naïve and sham groups. On the contrary, zerumbone-treated groups presented a significantly increased expression in comparison to sham and vehicle groups.

Over-expression of NMDA NR2B subunit has also been reported in the brain and spinal cord in chronic pain conditions (Tang et al., 1999; Wei et al., 2002). The increased expression of NR2B subunit is linked to inflammation leading up to persistent pain, where its over-expression was observed in the ACC in the Complete Freund’s Adjuvant chronic inflammatory animal model (Wu et al., 2005). The use of CCI model as well as whole brain sample could be a possible rationalization to the insignificant change in NMDA NR2B subunit observed in this study. Furthermore, previous studies have reported a down-regulation of the NR2B subunit in analgesic compounds (Hu et al., 2009; Wang et al., 2013). It is reasonable to hypothesize that the up-regulation observed in zerumbone-treated groups is due to the acute administration of treatment— which may not be sufficient to cause any effect of NR2B expression in the brain. Possibility of a modulatory action of NR2B subunit up-regulation should be further explored.

It is interesting to note that the expression of α_2A_-AR was up-regulated while both the TRPV1 and NMDA NR2B receptors were down-regulated respectively in the *in vitro* SH-SY5Y neuroblastoma cell model. These findings are coherent with both the behavioral allodynia and hyperalgesia assays. We note that the etiology of neuropathic pain is complex, and in some cases findings are contradictory (Lee et al., 2013) and left unexplained, however in this case, we postulate that zerumbone interacts and possibly triggers modulation differently in the peripheral nervous system compared to the central nervous system. As we have explained the proposed mechanisms in the brain regions explicitly in the preceding paragraphs, we can only conclude that in the peripheral nervous system, zerumbone acts as an agonist for the α_2A_-adrenoceptor (Kimura et al., 2012), and modulates both the TRPV1 (Perry et al., 2007; Yamamoto et al., 2008) and NMDA NR2B (Hu et al., 2009; Wang et al., 2013) receptors. These receptors and pathways are well established and studied.

Our findings on the expression of α_2A_-adrenoceptor, TRPV1 and NMDA NR2B receptors at the brain regions complemented by the exact opposite on the *in vitro* model further reiterated the plasticity of neuronal signaling in pain and the nervous system. While we know that the dorsal horn is one of the first point for pain signal transmission, a huge body of literature is now pointing to the relevance of the descending control of the nervous system in further regulating and modulating pain signals both ascending and descending. Interestingly, they can be both facilitatory or inhibitory or both (Heinricher et al., 2009).

In conclusion, our findings indicate the interaction between the noradrenergic system, TRPV1, NMDA receptors, and zerumbone in exhibiting anti-allodynic and antihyperalgesic effects in a neuropathic pain mice model. Moreover, the action of zerumbone on α_2A_-adrenoceptor, TRPV1 and NMDA NR2B receptor expression provides significant information on the mechanism of action of zerumbone. In support with previous studies on zerumbone against neuropathic pain, zerumbone has high potential as an antinociceptive compound for treatment of neuropathic pain. Research into new and better treatments for neuropathic pain patients are in critical need. A combinatorial therapy approach, consisting of drugs with different mechanisms of action, is currently used to treat neuropathic pain patients. Future research into the effect of zerumbone, in both chronic and acute treatments, on the relationship between various pain modulatory pathways in neuropathic pain models should be conducted.

## Data Availability Statement

The datasets used and/or analysed for this manuscript are available from the corresponding author on reasonable request.

## Ethics Statement

The animal study was reviewed and approved by the Institutional Animal Care and Use Committee (IACUC) UPM (Ref: UPM/IACUC/AUP- R060/2013).

## Author Contributions

All authors equally contributed to the study and critically reviewed the final version of the manuscript

## Funding

This study was supported by the Putra Grant—Putra Graduate Initiative (GP-IPS/2017/9528400), the Putra Grant—High Performance Research Grant (GPB-9659000) from Universiti Putra Malaysia and the Science Fund Scheme (Grant No: 5450778) under the Ministry of Science, Technology & Innovation Malaysia.

## Conflict of Interest

The authors declare that the research was conducted in the absence of any commercial or financial relationships that could be construed as a potential conflict of interest.
