# Supplementary material for: Zerumbone Modulates α2A-Adrenergic, TRPV1, and NMDA NR2B Receptors Plasticity in CCI-Induced Neuropathic Pain In Vivo and LPS-Induced SH-SY5Y Neuroblastoma In Vitro Models
Source: Front Pharmacol. 2020 Mar 4;11:92. doi: 10.3389/fphar.2020.00092 (PMC7064019; doi:10.3389/fphar.2020.00092)
Supplement: Supplementary file 1 [file DataSheet_1.pdf]

Supplementary material

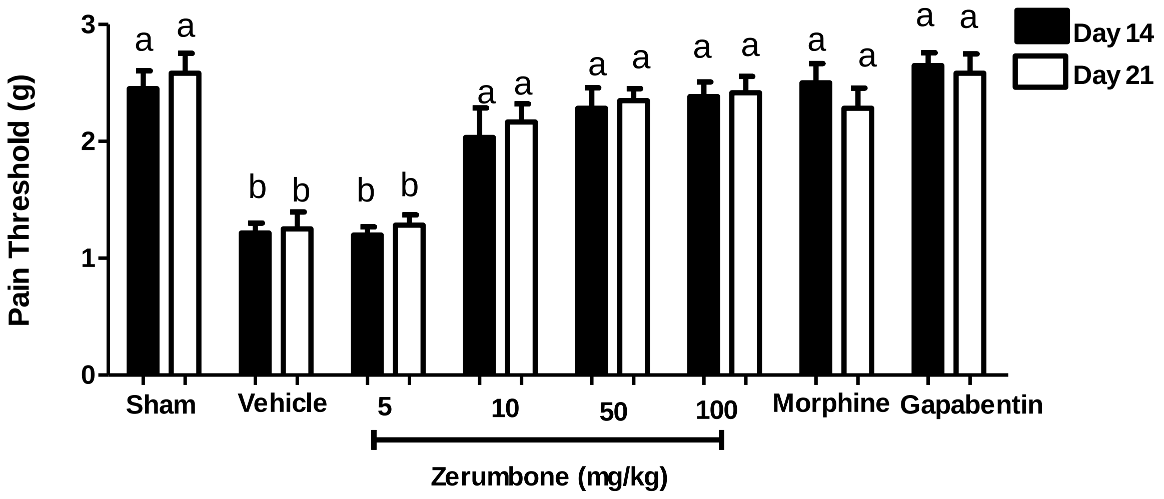

Fig. S1. Effects of Zerumbone on CCI-induced neuropathic pain in mice on day 14 and day 21 using the von Frey filament assessment for the ipsilateral paw. Each column represents the mean ± SEM of six mice. Statistical analysis was determined by one-way ANOVA followed by Tukey's post-hoc test with values of different superscript letters (a,b) as statistically different from each other (p<0.05).

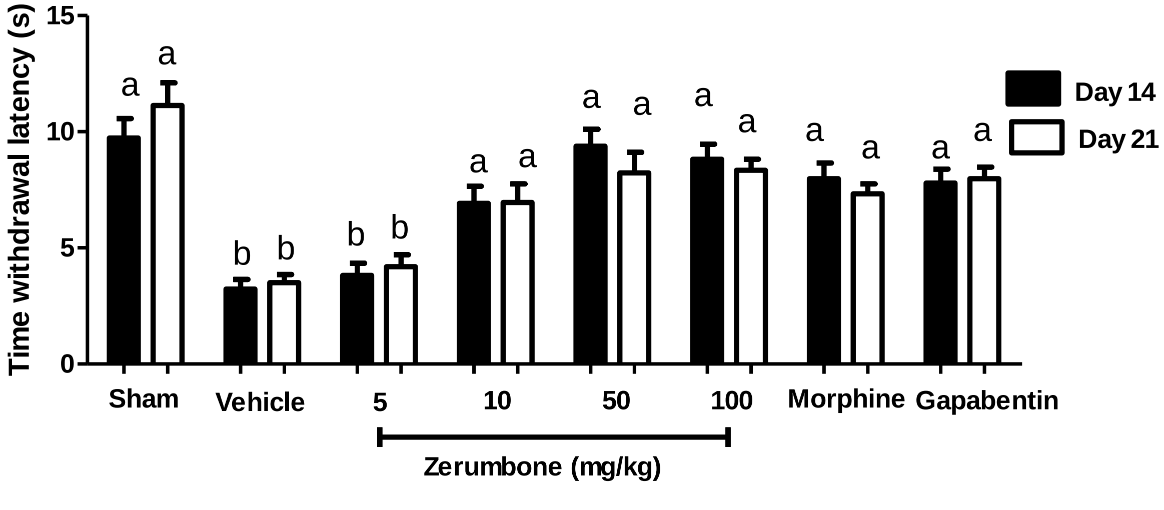

Fig. S2. Effects of Zerumbone on CCI-induced neuropathic pain in mice on day 14 and day 21 using the Hargreaves plantar test assessment for ipsilateral paw. Each column represents the mean ± SEM of six mice. Statistical analysis was determined by one-way ANOVA followed by Tukey's post-hoc test with values of different superscript letters (a,b) as statistically different from each other (p<0.05).

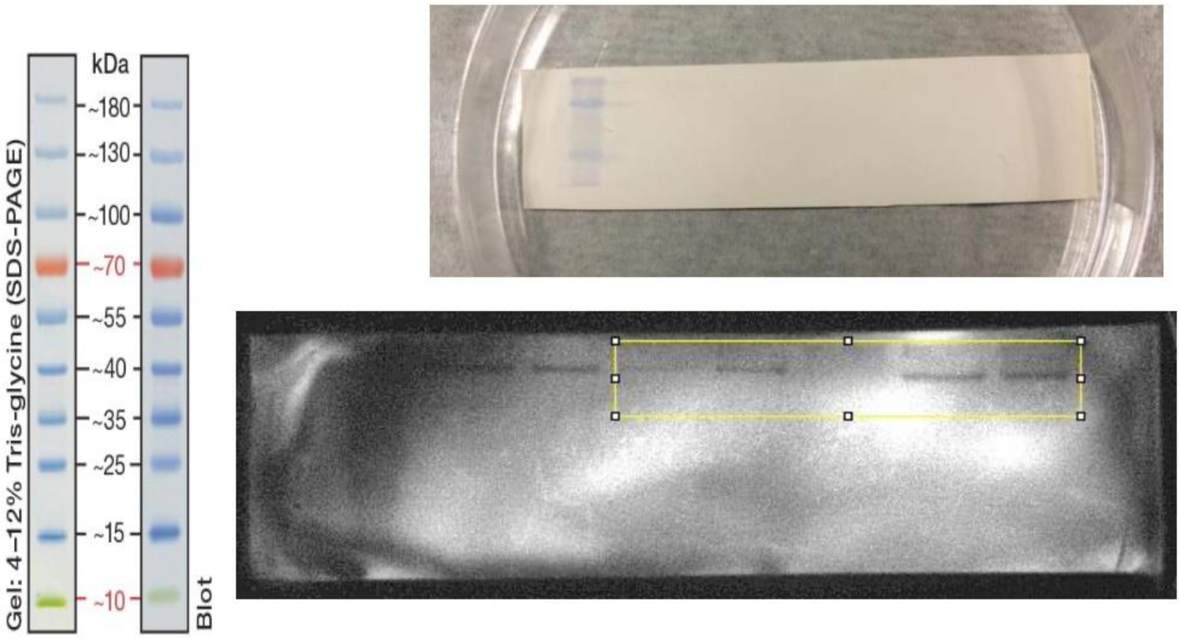

Fig. S3. Full image scan of α_{2A}-adrenergic receptor blot with ladder on the left lane. Band to correspond to ~48 kDa.

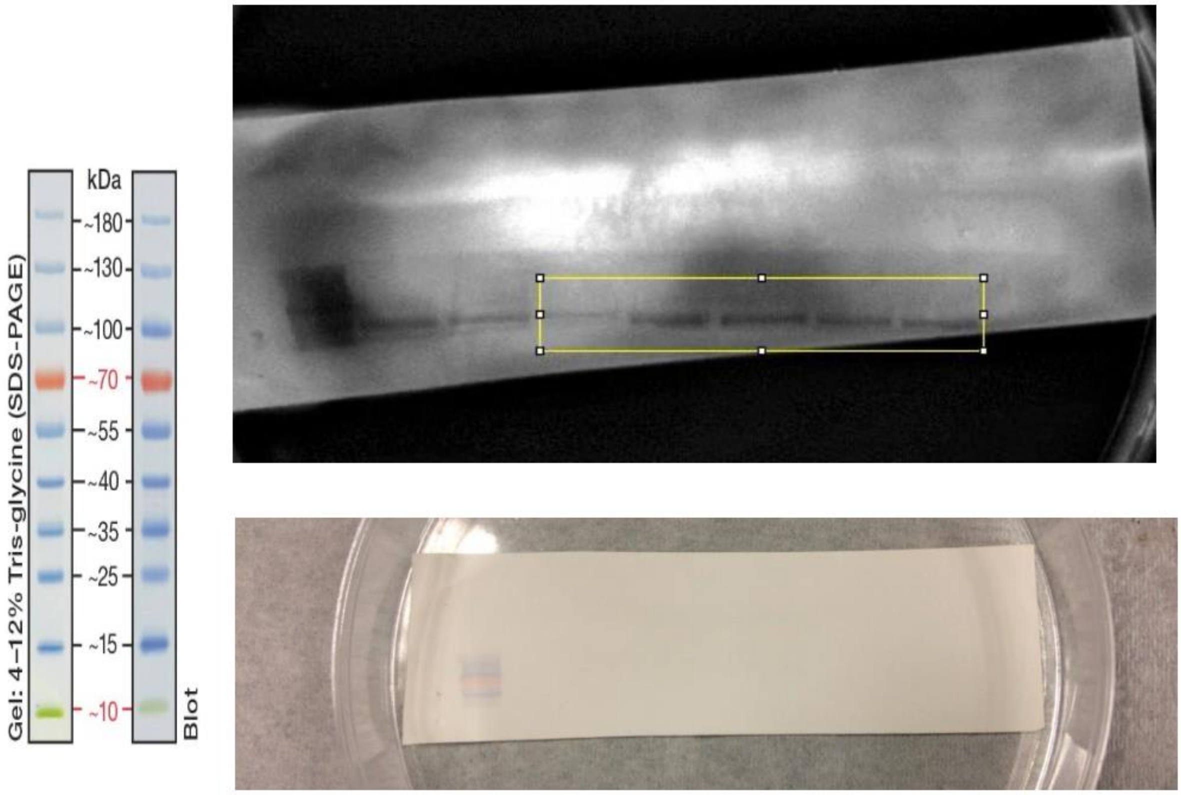

Fig. S4. Full image scan of TRPV1 receptor blot with ladder on the left lane. Band to correspond to ~92 kDa.

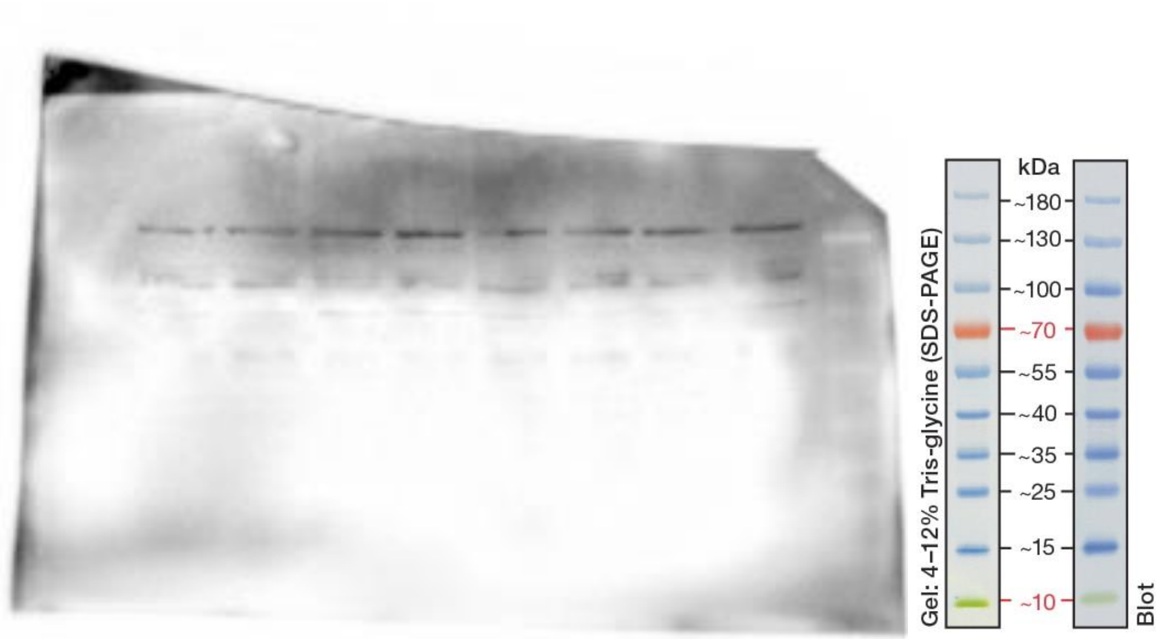

Fig. S5. Full image scan of NMDA NR2B receptor blot with ladder mildly visible on the right most lane. Band to correspond to ~160 kDa.

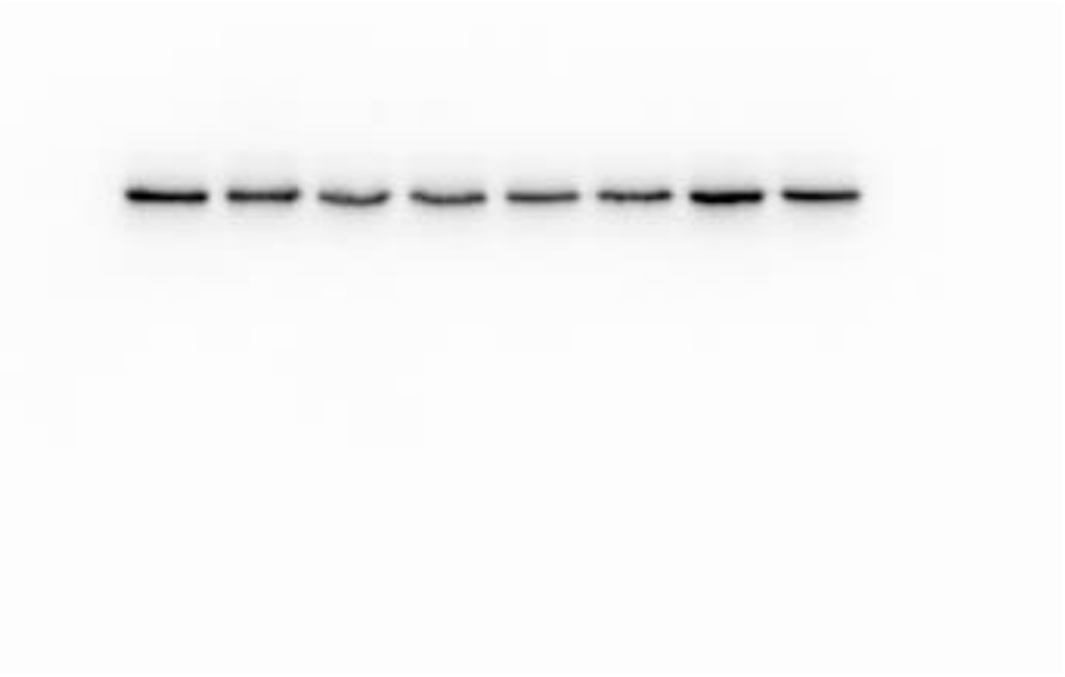

Fig. S6. Full image scan of β-actin blot.

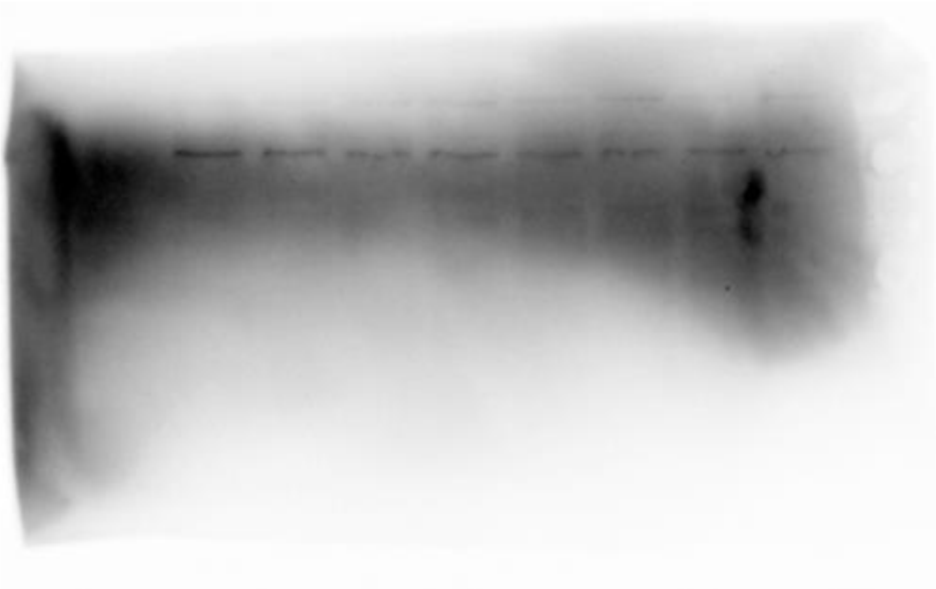

Fig. S7. Full image scan of α_{2A}-adrenergic receptor blot.

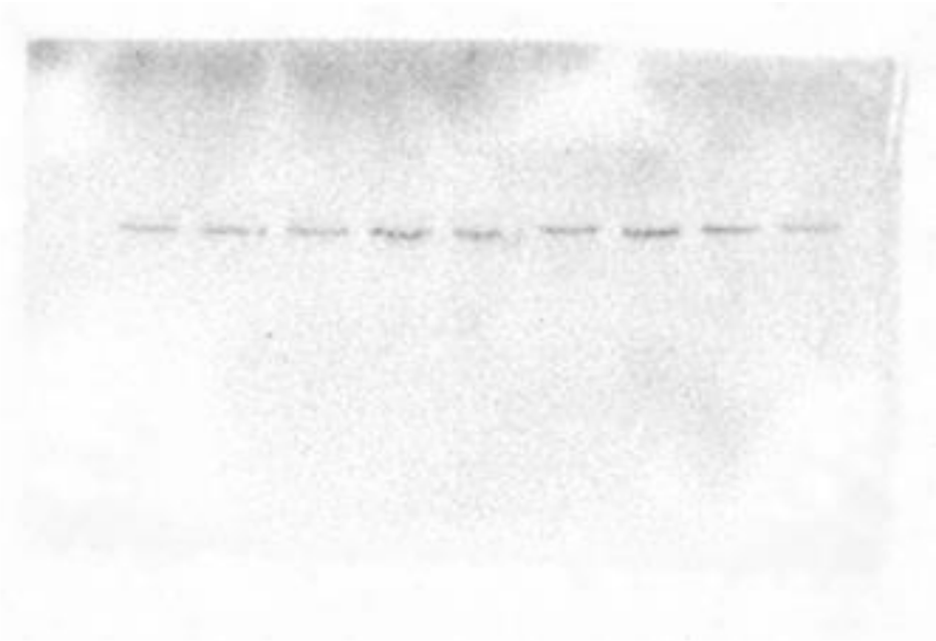

Fig. S8. Full image scan of TRPV1 receptor blot.

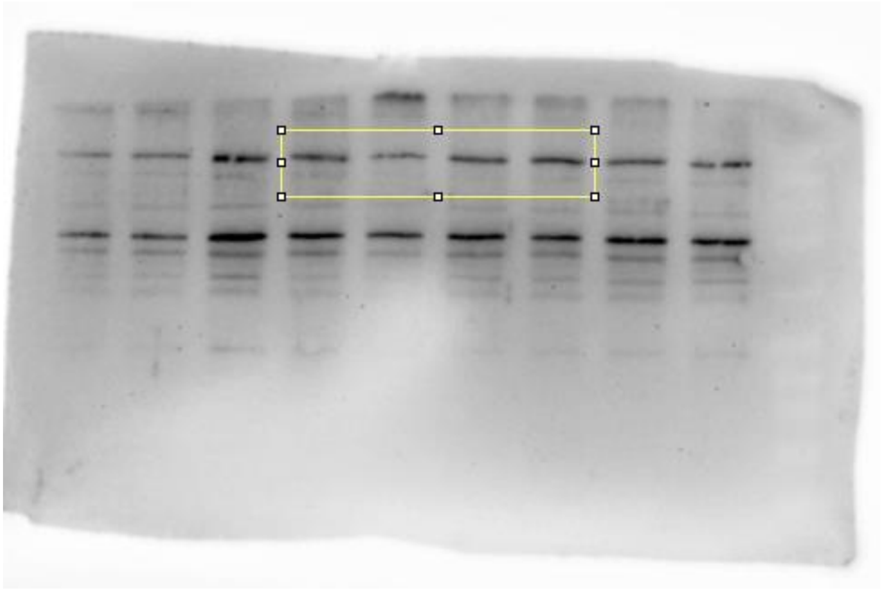

Fig. S9. Full image scan of NMDA NR2B receptor blot.
